# A review on natural enzyme inhibitors: an effective approach to control postprandial hyperglycemia in type II diabetes

**DOI:** 10.1007/s13659-026-00606-2

**Published:** 2026-07-13

**Authors:** Faezeh Shirkhan, Saeed Mirdamadi, Behrouz Akbari-adergani, Hadis Aryaee

**Affiliations:** 1 Department of Food Science and Technology, Faculty of Pharmacy, Tehran Medical Sciences, Islamic Azad University, Tehran, 19496-35881 Iran; 2 Department of Biotechnology, Iranian Research Organization for Science & Technology (IROST), Tehran, 33131-93685 Iran; 3 Water Safety Research Center, Food and Drug Administration, Ministry of Health and Medical Education, Tehran, 11136-15911 Iran; 4 Department of Biotechnology, Iranian Research Organization for Science & Technology (IROST), Tehran, 33131-93685 Iran

**Keywords:** Diabetes, Natural enzyme inhibitor, Bioactive peptides, Phytochemicals, Probiotics, Food

## Abstract

Type II diabetes (T2D) is a chronic disease caused by hyperglycemia, insulin resistance, and insufficient insulin production. Inhibition of α-amylase, α-glucosidase, and dipeptidyl peptidase (DPP-IV) enzymes is considered a crucial interventional approach in managing T2D. Therefore, identification of natural enzyme inhibitors is essential. Hence, this study aims to highlight existing gaps and future needs regarding the potential application of natural inhibitors such as carbohydrates, lipid compounds, phytochemicals, probiotic strains, and bioactive peptides in the inhibition of enzymes involved in T2D as well as their inhibitory effects which were reviewed completely. The results of most studies showed that phenolic compounds and polysaccharides impart significant biological potential in inhibiting enzymes. This review highlights that food products fermented by probiotics possess considerable potential for diabetes control. Peptides, liposoluble, and phytochemical compounds play an obvious role in diabetes management, promising results from enzyme inhibition assays suggesting their potential as safer alternatives to conventional treatments for diabetes. However, several limitations such as type of assay methods, enzyme sources, bioavailability, and lack of research resources were observed. Therefore, additional studies should be carried out to establish optimal dosages, bioavailability, and validation methods. Understanding the inhibitory mechanisms of natural enzyme inhibitors and examining the structure–activity relationships between them are critical subjects that must receive more attention in future researches. Additionally, it is important to address the gap between scientific research and the commercialization of natural products. Hence, future efforts should prioritize conducting cell-based studies and clinical trials to evaluate the efficacy and safety of these products.

**Graphical Abstract:**

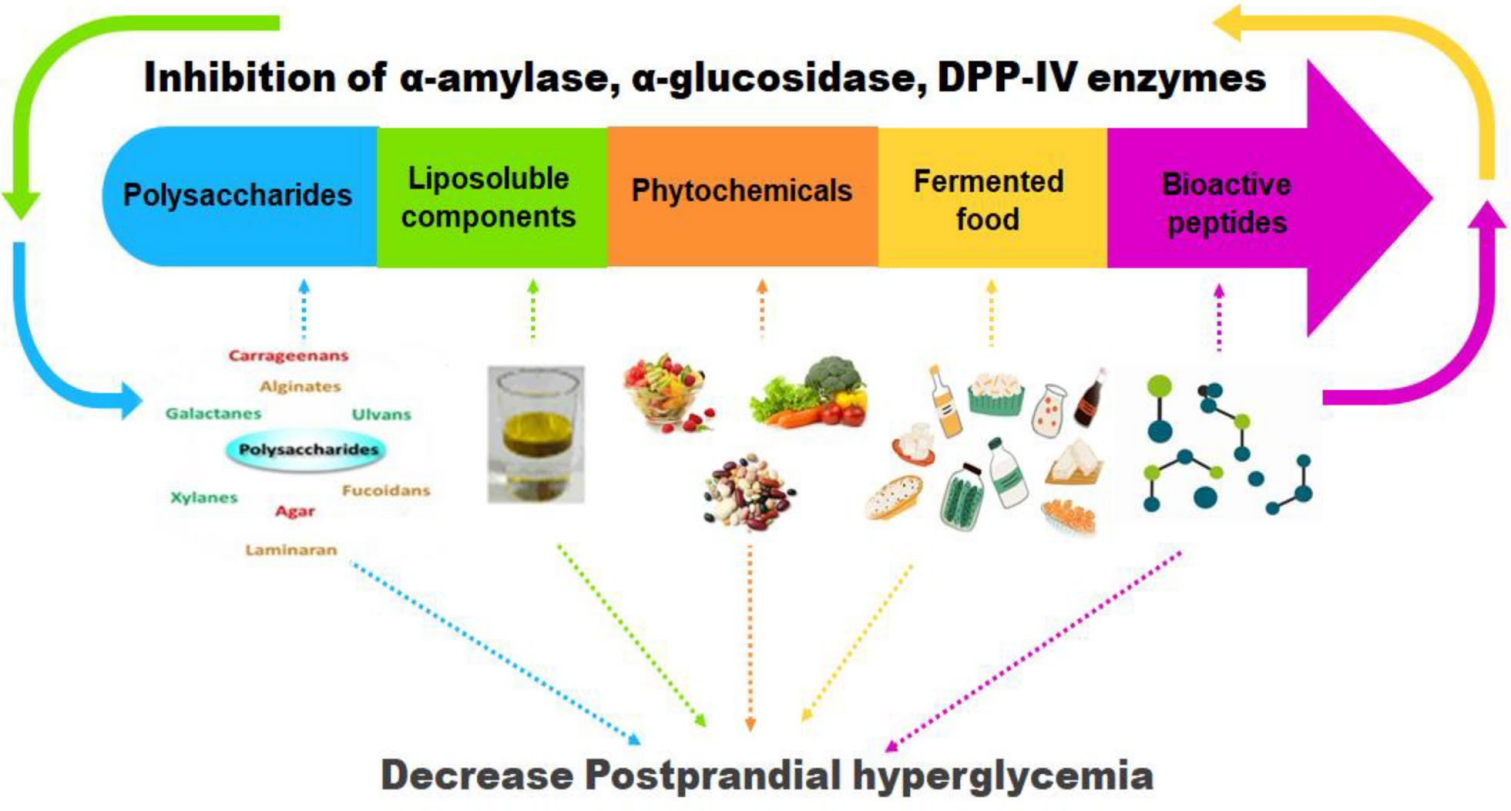

## Introduction

Diabetes mellitus is a metabolic disorder characterized by hyperglycemia resulting from deficiencies in insulin secretion, impaired insulin action, or insulin resistance [1]. Type II diabetes (T2D) is associated with hyperglycemia and insulin resistance [2]. A rapid rise in blood glucose levels is caused by the hydrolysis of starch by pancreatic α-amylase and the subsequent absorption of glucose by intestinal α-glucosidase [3] and dipeptidyl peptidase-IV (DPP-IV) [4]. Consequently, the blood glucose levels in diabetic patients are significantly higher compared to those of non-diabetic individuals [5]. Currently, the medical management of diabetes using drugs such as acarbose, meglitinides, biguanides, thiazolidinediones, and sulfonylureas imposes substantial financial burdens on patients. These medications, considered the primary agents for controlling postprandial hyperglycemia, have effectively lowered blood sugar levels [6]. However, they often lead to adverse effects, including digestive issues, weight gain, bloating, abdominal pain, and diarrhea, and their efficacy tends to diminish over time [7]. Recently, there has been increasing interest in identifying more effective and sustainable anti-hyperglycemic agents. For this reason, developing natural approaches for new diabetes treatments has gained attention [8]. At present, the use of alternative therapies instead of drugs and the use of functional foods have attracted the attention of consumers [9]. This growing need has emphasized exploring natural inhibitors with minimal side effects derived from food matrices [10]. The natural inhibitors with hypoglycemic property have fewer potential adverse effects and more efficacy than hypoglycemic agents [11]. Therefore, they may be good alternatives to anti-diabetic pharmaceuticals. In this regard, studies have shown that some carbohydrates have an effect on diabetes as enzyme inhibitors [12]. Some findings showed that oils and liposoluble compounds also strongly inhibited these enzymes [13]. Many studies demonstrated that phytochemicals have a glycemic-lowering effect [14]. Some research shows that probiotics and their metabolites such as bioactive peptides have anti-diabetic activity [15]. Such compounds may have a role in the treatment of diabetes, and when combined with other hypoglycemic drugs, they can result in a more significant decrease in blood sugar. However, there are limited comprehensive studies about these compounds that inhibit α-amylase, α-glucosidase, and DPP-IV enzymes. Given the significance of utilizing natural inhibitors as functional ingredients, supplements, or alternatives to pharmaceuticals for delaying or reducing glucose absorption in diabetic patients, this study focused on identifying such inhibitors for diabetes management. Hence, the main purpose of this review is to focus on the advances of natural inhibitors and introduce the natural products that affect these enzymes. In this context, carbohydrates, liposoluble compounds, phytochemicals, fermented food products produced by probiotics, and bioactive peptides are evaluated for their enzyme inhibition and potential applications in diabetes management. Also, the efficiency of inhibitory activity of these compounds on α-amylase, α-glucosidase, and DPP-IV enzymes is discussed.

## Methodology

A systematic literature search was performed to assess the antidiabetic properties of bioactive compounds and their inhibitory effects on α-amylase, α-glucosidase, and DPP-IV enzymes, using targeted keywords including “diabetes”, “bioactive peptides”, “probiotics”, “carbohydrates”, “lipophilic components”, “phytochemicals”, “polysaccharides”, and “functional foods”. Relevant studies were retrieved from major scientific databases, including PubMed, Springer, Science Direct, Scopus, Google Scholar, and Web of Science Core Collection. Extracted data were synthesized into Tables 1, 2, 3, 4, 5 and subjected to comparative analysis for interpretative evaluation.

**Table 1:** Inhibitory activity of carbohydrate (especially polysaccharide) by α-amylase and α-glucosidase enzymes

Type of polysaccharide	Sample	Source	Enzyme	IC_50_	Refs.
Neutral polysaccharide	Barley polysaccharides	Seed of barley	α-glu	22.49^a^	[29]
	Corn silk polysaccharides	Carboxyl methylated Sulfated Raw polysaccharide Acetylated	α-amy	5.33^a^ 8.54^a^ 10.07^a^ 10.31^a^	[30]
	Neutral polysaccharide (APPS1-2)	Apricot (*Armeniaca sibirica L. Lam*.) pulp	α-glu	6.06^a^	[31]
	Neutral polysaccharide	Seed of Fagopyrum tartaricum	α-glu	–	[32]
	Sulfated polysaccharides	Marine algal- *Cystoseira crinita*	α-amy	0.04^a^	[33]
	Polysaccharide (4860 kDa)	Moringa oleifera Lam. leaves	α-amy	–	[34]
	Buckwheat, or oats, wheat gluten	Wheat Aleurone & Bran	α-amy	0.14^a^ 0.023^a^	[35]
	Erythritol Attenuates	Grapes, watermelon, pears	α-glu	0.053^b^	[36]
	Neutral polysaccharide	*Glycyrrhiza* residue	α-glu	0.40^a^	[37]
	Neutral polysaccharide AWPA	*Annona squamosa* residue	α-amy α-glu	1.40^a^ 0.70^a^	[38]
Sulfated polysaccharides	Fucoidan	Fucusvesiculosus	α-glu	0.05^a^	[39]
		Ascophyllum nodosum (ANF)	α-amy α-glu	0.12 ∼ 4.64^a^ 0.01∼0.05^a^	[39]
		Turbinaria ornate	α-amy	0.034^c^	[40]
		Sargassum wightii	α-glu	0.033^c^	[41]
		Brown seaweed Saccharina japonica	α-amy	0.20^a^	[42]
Plant polysaccharide (dietary fibers)	Cellulose	–	α-amy	2.80^a^	[28]
	Pectin	Ectract pectin from pomegranate peel (Buffer, enzymatic, acid)	α-amy	Buffer: 0.28^a^	[43]
				Enzyme: 0.12^a^	
				Acid: 1.96^a^	
			α-glu	Buffer: 1.23^a^	
				Enzyme: 4.02^a^	
				Acid: 10.22^a^	
	Commercial pectin	Citrus peel pectin	α-amy	–	[44]
			α-amy α-glu	– 2.38 ^a^	[45]
	β-glucan	Yeast cell walls	α-amy α-glu	– –	[46]
	Feruloylated arabinoxylan mono- & oligosaccharides	Corn bran & wheat aleurone	α-glu	1.03–1.65^a^ and 2.60–6.50^a^ on Caco-2 cells and rat intestinal, respectively	[47]
Others	Alginates	Marine algal- *Laminaria digitate*	α-amy	0.10^a^	[48]

α-Amylase (α-amy), α-Glucosidase (α-glu)

^a^IC_50_ values are expressed as mg/mL

^b^IC_50_ values are expressed as mM

^c^IC_50_ values are expressed as mg

**Table 2:** Inhibitory potential of liposoluble components from oil on glycemia regulating enzyme

Type of liposoluble components	Source	Inhibitors	Enzyme	IC_50_	Refs.
Fatty acid	Seed oil	Methyl linoleate	α-glu	51.80^b^	[13]
		Methyl steareate	α-glu	24.80^b^	
		Methyl oleate	α-glu	20.10^b^	
		Stearic acid	α-glu	22.20^b^	
		Oleic acid	α-amy	0.10^a^	
			α-glu	0.064^a^	
		Palmitic acid	α-amy	0.04^a^	
			α-glu	21.30^b^	
		Linoleic acid	α-amy	0.023^a^	
			α-glu	0.074^a^	
	–	10-hydroxy-8(E)-octadecenoic acid	α-glu	70.00^b^	[62]
Phytosterols	*Chrozophoraplicata*	β-sitosterol	α-glu	287.10^b^	[60]
Terpenes	Winged bean	Betulinic acid	α-glu	10.60^b^	[63]
	Banana peel	Cycloeucalenone	α-amy	20.30^b^	[64]
			α-glu	31.80^b^	
		31-norcyclolaudenone	α-amy	27.60^b^	
			α-glu	38.80^b^	
Wheat	Bran	1,2-Dilinoleylglycerol-3 phosphate	α-glu	38.90^b^	[65]
		1-Palmitoyl-2-linoleoyl glycerol-3-phosphate	α-glu	47.90^b^	
	Bran & germ	1,2-Dilinoleylglycerol-3-phosphate	α-glu	0.03^a^	[66]
		1-Palmitoyl-2-linolery glycerol-3-phosphate	α-glu	0.032^a^	
	Bran	Alkylresorcinols	α-glu	0.04^a^	[67]
		1,2-Dilinoleylglycerol-3-phosphate	α-glu	38.90^b^	[65]
		1-Palmitoyl-2-linoleoyl glycerol-3-phosphate	α-glu	47.90^b^	
Fat-soluble vitamins	*Cosmoscaudatus* Leaves	Vitamin E	α-glu	–	[68]

α-Amylase (α-amy), α-Glucosidase (α-glu)

^a^IC_50_ values are expressed as mg/mL

^b^IC_50_ values are expressed as µM

**Table 3:** Inhibitory activity of phytochemicals on α-amylase, α-glucosidase, and DPP-IV enzymes

Type of foods	Sample	Active phytochemical	Inhibitor	Enzyme	IC_50_	Refs.
Fruits	Grape seed	Procyanidins (flavonoid)	Grape seed-derived procyanidins	DPP-IV	–	[72]
	*Vaccinium* spp. (blueberry)	Polyphenols Anthocyanins	Peel and flesh of blueberry	α-glu	–	[73]
	Aronia juice	Cyanidin	Aronia juice	DPP-IV	–	[74]
	Different citrus fruits from Aceh	Carotenoids	Peel and pulp	α-amy	–	[75]
	Peel of araticum fruit (*Annona crassiflora Mart*.)	Chlorogenic acid, catechin, procyanidins, caffeoyl-hexosides, quercetin-glucosides & kaempferol	Ethyl acetate and *n-*butanol fractions	α-amy α-glu	0.0045–0.0017^a^ 0.55–0.79^a^	[76]
	Foods (black tea, green tea, blueberry, blackberry, red cabbage, broccoli, black turtle bean, black soybean and legumes	Phenolic substances	Myricetin from black turtle bean	α-amy α-glu	0.40^a^ 0.00087^a^	[77]
	Citrus bioflavonoid standards and three Citrus bioflavonoid supplements bioflavonoids	Flavonoid	(rutin) (hesperitin and eriodictyol)	DPP-IV	485–5700^b^	[78]
	Pomegranate *Punica granatum* L	Phenolic acids Tannins	Extract and its fractions (petroleum ether, ethyl acetate, butanol and water) of Pomegranate	α-amy α-glu	Between 0.024 and 0.29^a^ Between 0.00026–0.0046^a^	[79]
	Aronia, bilberry, blackcurrant, cranberry, elderberry, lingonberry, pomegranate, red grape, and sour Cherry	Anthocyanins	Aronia Pomegranate Red grape	α-amy α-glu	0.33^a^ 0.80^a^ 0.65^a^ 0.12^a^ 0.13^a^ 0.16^a^	[80]
	Bilberry extract	Anthocyanins	Anthocyanin-rich bilberry extract	α-amy α-glu	4.06^a^ 0.31^a^	[81]
	Mulberry (*Fructus mori*)	Anthocyanins	Mulberry anthocyanins	α-amy α-glu	–	[82]
Vegetables	Eggplant (*Solanummelongena*)	Polyphenols	Eggplant phenolics	α-amy α-glu	–	[83]
	Onion (*Allium cepa L)*	Polyphenols Quercetin	Bulb (edible part) and outer fleshy layers and dry peels (inedible part)	α-amy α-glu	– –	[84]
	Different pepper cultivars at two stages of fruits ripening (immature and mature)	Polyphenols Flavonoids Carotenoids Capsaicinoids	Fiesta, Orange Thai, and Cayenne Golden cultivars	α-amy α-glu	Lipophilic fraction ranging from 9.1^a^ to 28.6^a^ in the immature stage –	[85]
	Greek oregano (*Origanum vulgare*), marjoram (*Origanum majorana*), rosemary (Rosmarinus officinalis), Mexican oregano (*Lippia graveolens*)	Phenols and flavonoids	Greenhouse rosemary Mexican oregano marjoram extract	DPP-IV DPP-IV DPP-IV	0.02^b^ 0.03^b^ 0.06^b^	[86]
	Lotus	Flavonoids	Lotus leaf flavonoids	α-amy	5.60^a^	[87]
	Polyphenolic extracts from oregano species	Polyphenols	*Hedeoma patens, Lippia graveolens Lippia palmeri*	α -amy α-glu	– –	[8]
	Purple sweet potato (*Ipomoea batatas* L.)	Anthocyanin	Diacylated anthocyanins	α-amy α-glu	0.08^a^ 1.60^a^	[88]
	*Thymus comosus* Heuff. ex Griseb. et Schenk (*wild thyme*)	Anthocyanin	Polyphenol-enriched extract from the sample	α-glu	1.99^a^	[89]
Cereals & whole grains	Germination of seven selected commercially important grains	Phenolic compound	Germinated sorghum rye extracts	α-amy α-glu	– –	[90]
	Black gram (*Vigna mungo L.*) and its milled by-products	Phenolic acids	Black gram flour and its milled fractions	α-glu	–	[91]
	Black legumes, black soybean (*Glycine max*), black turtle bean	Phenolic substances (myricetin)	Fraction V from black soybean and turtle bean	α -amy	Black soybean: 0.25^a^	[92]
				α-glu	black turtle bean: 0.002^a^	
	Rice (*Oryza sativa L.* subsp. indica Kato, Saiyar Rice Co. Ltd, Xiangfan, China)	Anthocyanin	Anthocyanin from black rice starch extract	α-amy α-glu	0.70^a^ 1.30^a^	[93]
	Purple corn extract (*Zea mays L.*)	Anthocyanin	Phenolics-rich extract	α-glu	–	[94]
Others	Herbal teas from Eastern Anatolia	Phenolic compound	*M. neglecta* *P. lanceolate* *V. cheiranthifolium* *A. elichrysifolium* *P. armeniaca*	α-amy α-glu	– 13.83^a^ 1.43^a^ 2.03^a^ 12.53^a^ 2.54^a^ 3.30^a^	[95]

α-amylase (α-amy), α-glucosidase (α-glu)

^a^IC_50_ values are expressed as mg/mL

^b^IC_50_ values are expressed as mM

**Table 4:** Recent cases reported on fermented foods with anti-diabetic properties

Type of fermented foods	Type of Strains	Enzyme	Result	Refs.
Yogurt	*S. thermophilus*1275 &* 285,* *L. delbrueckii ssp. bulgaricus* 1092* &* 1368*,* *L.acidophil* 4461* &* 33200*,* *L. casei* 2607* &* 15286*,* *B. longum 5022*	α-glu	All strains could inhibit the α-glucosidase enzyme	[120]
Skim milk	*L. acidophilus* XA0145	α-glu	The results showed that pear juice with *L. Acidophilus* could help to reduce hyperglycemia linked to T2D	[121]
Pear juice	*L. casei* 2W*,* *L. rhamnosus* Z7	α-glu	*L. casei 2W* and *L. rhamnosus Z7* indicated the potency to inhibit α-glucosidase enzyme activity	[122]
Yogurt,	*L. acidophilus* CCFM6*,* *L. plantarum* CCFM47*, L.CCF*M232*,* *L. rhamnosus GG (LGG)*	α-glu	Soybean oligosaccharides enriched yogurt improved probiotic (CCFM6 and CCFM47) improved α-glucosidase inhibition	[123]
Functional foods	*L. plantarum* strains ZF06-*1,* ZF06-3*,*IF2-14 *and* 17	α-glu	The results showed that the strains could able to inhibit α-glucosidase	[124]
Protein powder	*L. plantarum* *L. helveticus*	α-amy α-glu	The fermentation of camu-camu combined with soymilk enhanced anti-hyperglycemic bioactives by inhibiting α-amylase and α-glucosidase enzymes	[125]
Set yoghurt	*L. reuteri-*KX881777*,* *L. plantarum*KX881772*,* *L. plantarum-*KX881779, *L. plantarum* DSM246*8*	α-amy α-glu	The inhibition rate of α-glucosidase in both milk types fermented by *Lc. K782* ranged between 30 and 40%	[126]
Soybean	Eight LAB strains	α-glu	Among strains, *L*. *mali* K8 showed higher inhibition than the other strains	[127]
Commercially in functional foods	*L. mali K8*	α-glu	The pumpkin fermented by *L. mali* K8 inhibited the α-glucosidase enzyme (95.89 ± 0.30%)	[128]
Camu-Camu (Myriciaria dubia Mc. Vaugh), Soy milk	*Enterococcus spp. Four E. faecium strains, one E. faecalis, and one E. durans*	α-amy ⍺-glu	Fish sausages fermented by *Enterococcus spp.* inhibited α-amylase activity (29.2–68.7%) and α-glucosidase activity (23.9–41.4%)	[129]
Whey of fermented milk	*E. faecalis* DPC5154	α-glu	Fermented milk DPC5154 demonstrated the ability to inhibit α-glucosidase activity by 33.41%	[130]
Kefir grains	*L. lactis* KX881782	⍺-amy ⍺-glu	The hypoglycemic activities of fermented camel sausages were greater than beef sausages due to their higher inhibition of α-amylase and α-glucosidase	[131]
Fermented pumpkin-based beverage	*L. fermentum* (M2), *L. fermentum* (M3), *L. fermentum* (M4), *L. fermentum* (M7), *L. rhamnosus* (M8), *L. rhamnosus* (M9), *L. fermentum* (M10), *L. fermentum* (M17), *L. helveticus* (V3), *L. casei* (NK9)	α-amy α-glu	*L. fermentum* M2 exhibited superior inhibitory activity against α-amylase (65.29%) and α-glucosidase (11.14%), while *L. fermentum* M7 demonstrated inhibition rates of 63.47% for α-amylase and 13.74% for α-glucosidase	[132]
Fermented fish sausages	*L. lactis, L. delbeurkii*	α-amy	*L. delbeurkii* has higher anti-diabetic properties than *L. lactis* in cow and buffalo milk	[133]
Fermented milk	Ten LAB strains	⍺-amy ⍺-glu	The results indicated that milk fermented with *L. helveticus* PTCC 1930 exhibited the highest α-amylase inhibitory activity (35%), while milk fermented with *L. paracasei* ssp. *paracasei* 1945 demonstrated the highest α-glucosidase inhibitory activity (50%)	[2]

α-amylase (α-amy), α-glucosidase (α-glu)

**Table 5:** Enzyme inhibitory activity of protein hydrolysates from foods

Type of food products	Source	Protein hydrolysates	Enzyme	Peptide(s) identified	IC_50_	Refs.
Dairy products	Camel milk	Trypsin	DPP-IV	FLQY FQLGASPY ILDKEGIDY ILELA LLQLEAIR LPVP LQALHQGQIV MPVQA SPVVPF	> 1.00^b^ > 1.00^b^ 0.35^b^ 0.72^b^ 0.178^b^ 0.09^b^ > 1.00^b^ 0.093^b^ 0.214^b^	[145]
	Goat milk (Casein)	Trypsin & chymotrypsin	DPP-IV	MHQPPQPL SPTVMFPPQSVL VMFPPQSVL INNQFLPYPY AWPQYL	0.35^b^ 0.68^b^ – 0.04^b^ –	[146]
	Camel milk	Alcalase, bromelain, & papain	α-amy	MPSKPPLL, KDLWDDFKGL	– –	[147]
	Egg white protein	Alcalase	α-glu	RVPSLM TPSPR DLQGK AGLAPY RVPSL DHPFLF HAEIN QIGLF	0.023*10^−3b^ 0.04 *10^−3b^ > 0.01*10^−3b^ > 0.01*10^−3b^ > 0.01*10^−3b^ > 0.01*10^−3b^ > 0.01*10^−3b^ > 0.01*10^−3b^	[148]
	Egg yolk	Pepsin	α-glu	VTGRFAGHPAAQ	0.37*10^−3b^	[148]
	Turtle egg Egg yolk	Trypsin, pepsin, chymotrypsin, thermolysin, and GI enzyme	DPP-IV	LPSW WLQL LPLF VPGLAL LVGLPL	0.29^b^ 0.27^b^ 0.46^b^ > 2.00^b^ 0.43^b^	[149]
	Chicken egg	Pepsin and trypsin	DPP-IV	ADF MIR FGR	16.83^b^ 4.90^b^ 46.20^b^	[150]
Bean products	Black bean	Proteinase K, pepsin, trypsin, papain, alcalase, flavourzyme, themolysin, and chymotrypsin	α-glu α-amy	TTGGKGGK AKSPLF WEVM	– – –	[151]
	Soybean	Pepsin and pancreatin	α-amy α-glu	-	α-amy: 1.70^a^ maltase & sucrose 3.73^a^ α-glu: 2.90^a^	[152]
Cereals products	Walnut	Alcalase, Trypsin, Neutrase, Protamex and Flavourzyme	α-amy α-glu	LPLLR	–	[153]
	Oats	Pepsin, trypsin, pancreatin, alcalase	DPP-IV	LQAFEPLR EFLLAGNNK	0.10^b^ –	[154]
	Wheat (Gluten)	Debitrase HYW20	DPP-IV	VPL WL	–	[155]
	Yellow field pea	Alcalase, pepsin, trypsin, chymotrypsin	α-amy	-	0.22^a^	[156]
	Quinoa	Pepsin & pancreatin	DPP-IV α-glu	GEHGSDGNV IQAEGGLT	–	[157]
	Quinoa	Pepsin and pancreatin	α-glu	DKKYPK	–	[157]
	Quinoa	Papain, ficin & bromelain	DPP-IV	MAF NMF HPF MCG	–	[158]
Seeds	Cumin seed	Protamex	α-amy	FFRSKLLSDGAAAAKGALLPQYW, RCMAFLLSDGAAAAQQLLPQYW, DPAQPNYPWTAVLVFRH	– – – – –	[159]
	Hemp seed protein	Alcalase	α-glu	–	–	[160]
	Oileseed	Oilseed protein hydrolysate	DPP-IV	–	–	[161]
	Soybean seed	Alkaline, Proteinase, Papain Trypsin, Pepsin	α-glu	LLPLPVLK SWLRL WLRL	0.24 *10^−3b^ 0.18*10^−3b^ 0.16*10^−3b^	[162]
	Camellia seed cake	Alcalase, flavourzyme, pepsin, and trypsin	α-glu	GHSLESIK, GLTSLDRYK	–	[163]
	Watermelon seed	Pepsin,Trypsin,alcalase	α-amy	–	0.15–0.23^a^	[164]
Meat products	Cooked meat	Amylase, pepsin, trypsin	DPP-IV	IPI WL LPL WI FL LW VL WM ML IP LP AL	0.0035*10^−3b^ 0.04*10^−3b^ 0.24*10^−3b^ 0.09*10^−3b^ 0.40*10^−3b^ 0.10*10^−3b^ 0.07*10^−3b^ 0.24*10^−3b^ 0.09*10^−3b^ 0.15*10^−3b^ 0.71*10^−3b^ 0.90*10^−3b^	[165]
	Chicken by product	Flavourzyme & corolase	DPP-IV	TL HT LA LADVEVDLL LL ETGKGEDGE FL LFFSMLLML LF	– – – – – – – – –	[166]
	Silver carp	Trypsin, neutrase, alcalase, papain, pepsin, & flavourzyme	DPP-IV	LPIIDI APGPAGP AGPPGPSG ALAPSTM	0.10^b^ 0.23^b^ – –	[118]
	Silver carp	Papain, bromelain, alcalase 2.4 L, neutrase, & flavourzyme	DPP-IV	WGDEHIPGSPYH IAQPQEKAPDPFRH IAGPAGPRGPAGPN VAPEEHPTL YALPHAI EPGNPGPAGPA	0.35^b^ 0.81^b^ 1.17^b^ 1.93^b^ 2.30^b^ 2.94^b^ – –	[167]
	Tuna	Protease XXIII, orientase 90N	DPP-IV	PGVGGPLGPIGPCYE CAYQWQRPVDRIR PACGGFWISGRPG	0.12^b^ 0.08^b^ 0.10^b^ –	[167]
	Bambara bean	Alcalase & thermolysin	DPP-IV	–	1.73^a^	[168]
	Chickpea	Pepsin, pancreatin bromelain	DPP-IV	PHPATSGGGL YVDGSGTPLT	0.24^a^ –	[169]
Others	Almond (Armeniaca sibirica) oil	Prote Ax and Protease M	α-glu	WH	0.02*10^−3b^ for both before and after digestion	[170]
				WS	Trp-Ser increased after simulated digestion, from 0.02 to 0.04*10^−3b^	
	Mulberry Leaves	Alcalase	α-glu	RWPFFAFM AAGRLPGY	1.30^b^ 1.32^b^	[171]

α-Amylase (α-amy), α-Glucosidase (α-glu)

^a^IC_50_ values are expressed as mg/mL

^b^IC_50_ values are expressed as mM

## Digestion of carbohydrate by enzyme inhibition

**Fig. 1:**
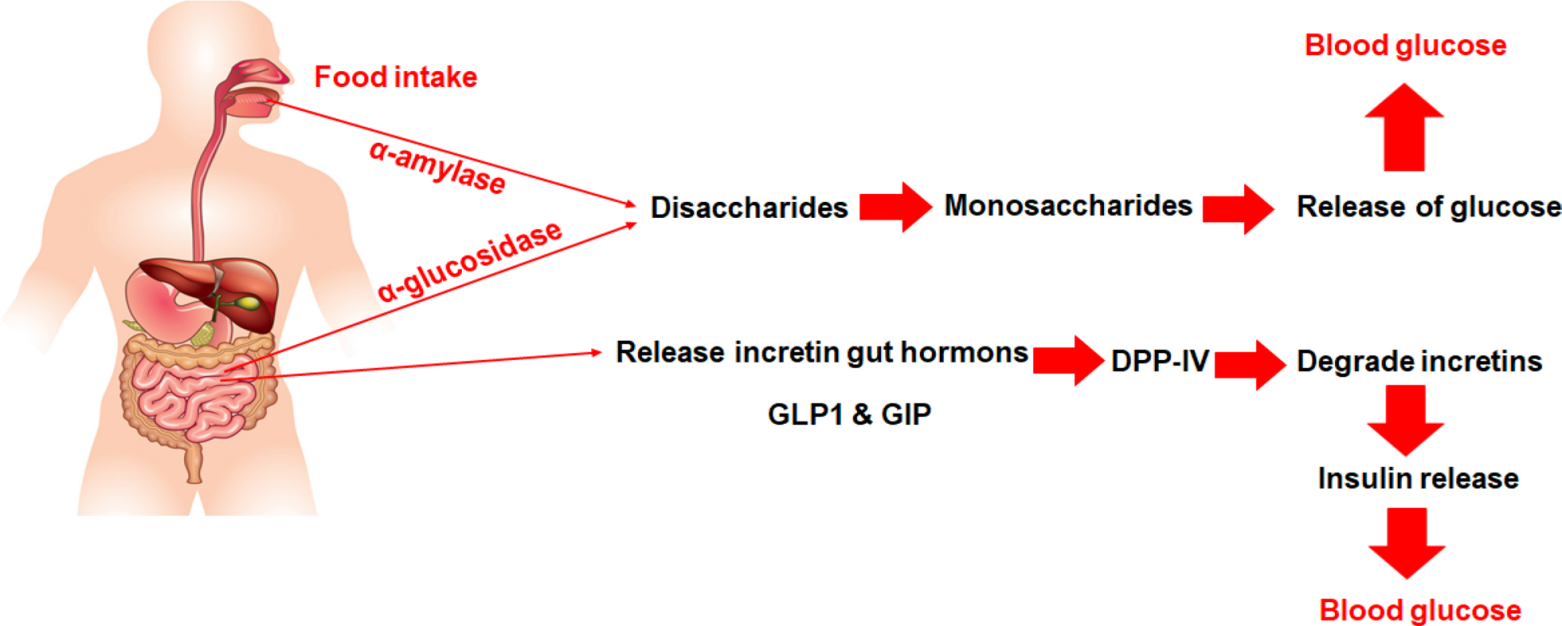
Action mechanism of α-amylase, α-glucosidase, and DPP-IV enzymes in the human Body

Different organs in the body (salivary glands, pancreas, and small intestine), enzymes, and proteins secreted from them allow the digestion of carbohydrates. Therefore, they affect the blood glucose level. Mechanisms of important enzymes in the human body are shown in Fig. 1.

According to Fig. 1, the α-amylase enzyme, produced by the salivary glands and pancreas, facilitates the hydrolysis of complex carbohydrates like starch and glycogen into simpler sugars such as glucose and maltose, which contributes to increased blood glucose levels [16]. Following this, the α-glucosidase enzyme in the small intestine breaks down disaccharides into glucose, highlighting its pivotal role in the digestion and metabolism of carbohydrates [17]. In a separate physiological process following food consumption, specialized endocrine neurons in the digestive system release peptides that enhance glucose metabolism and energy homeostasis. Notable among these intestinal hormones are incretins, including glucagon-like peptide-1 (GLP-1) and glucose-dependent insulinotropic peptide (GIP). These incretins stimulate meal-induced insulin secretion by the pancreas, suppress glucagon release, and improve glucose utilization. Conversely, it has been observed that DPP-IV effectively degrades GLP-1 and GIP. Since GLP-1 and GIP stimulate pancreatic insulin production, DPP-IV inhibitors play a critical role in preventing the degradation of these incretins, thereby enhancing insulin secretion and mitigating postprandial hyperglycemia [18].

## Inhibition of key enzymes by natural enzyme inhibitors linked to T2D

### Carbohydrate derivative inhibitors

**Fig. 2:**
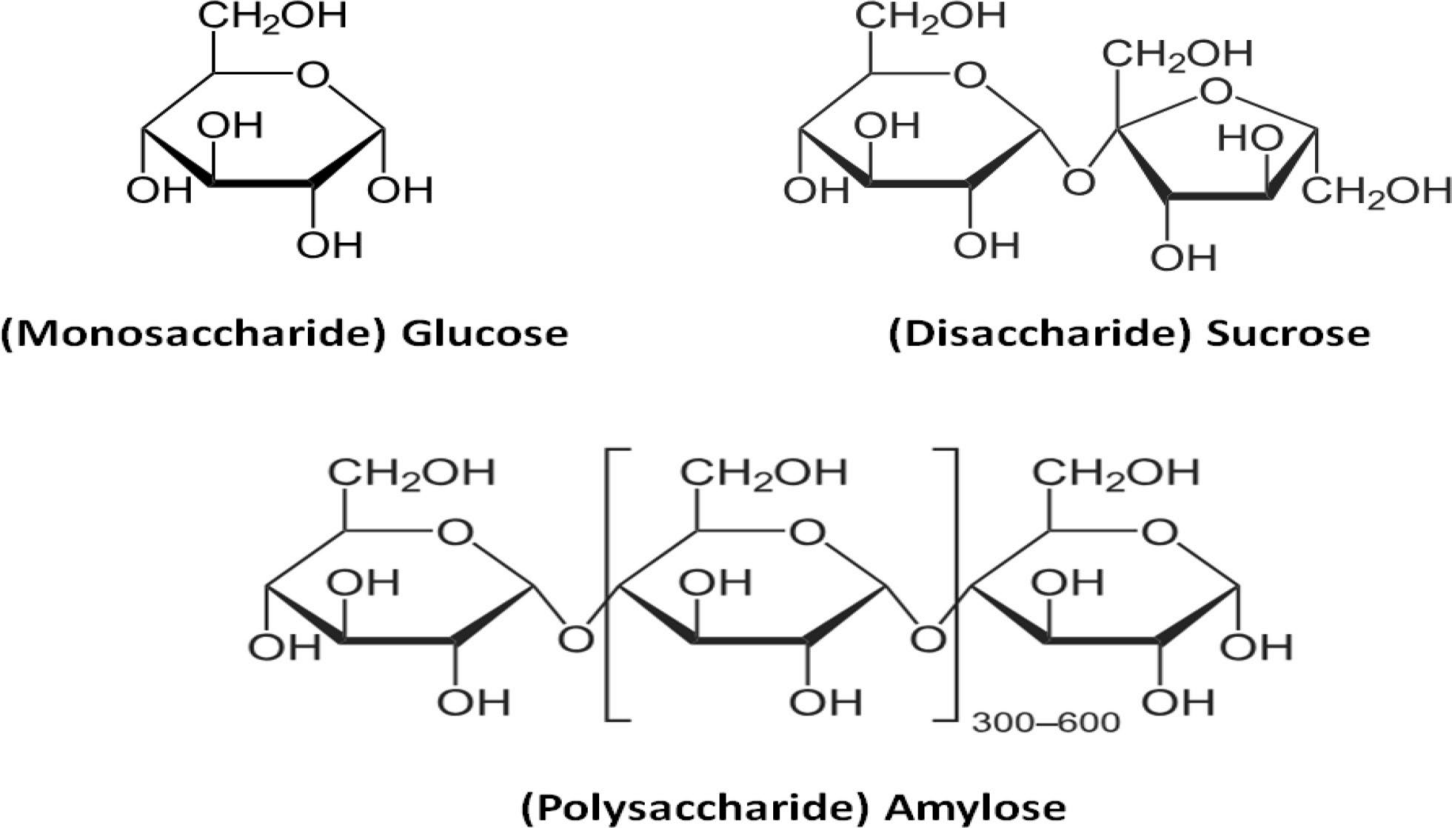
Molecular structures of carbohydrates for typical monosaccharide, oligosaccharide, and polysaccharides

Carbohydrates are the primary source of energy for the body. They are found in a wide array of foods. Carbohydrates come in a variety of forms [19]. Based on molecular structure, carbohydrates are classified as monosaccharides, oligosaccharides, and polysaccharides. The difference between their structures can be seen in Fig. 2.

**Fig. 3:**
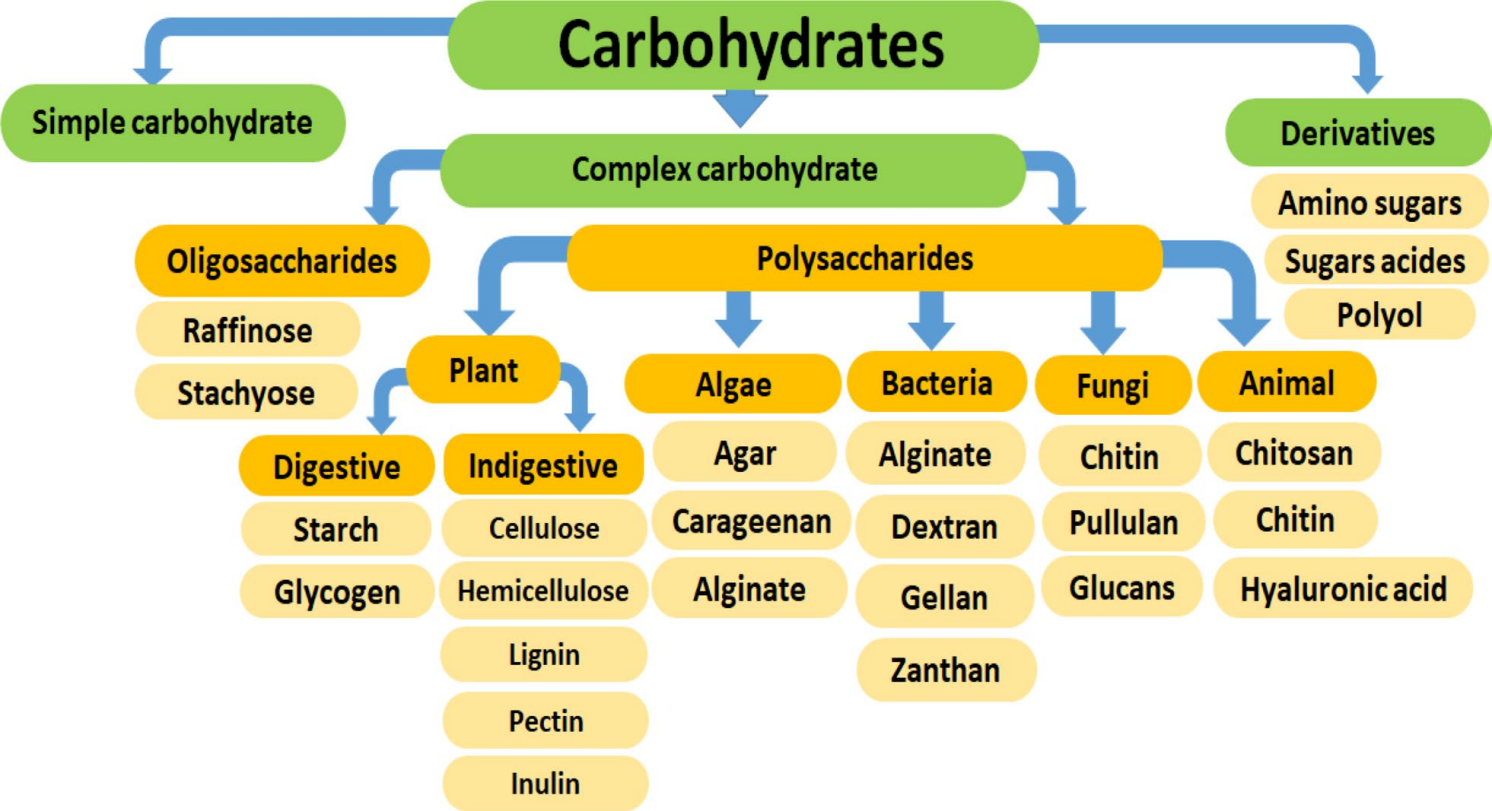
Classification of carbohydrates and compounds that could act as hypoglycemic agents

Carbohydrates can be divided into two main categories as simple and complex carbohydrates. Simple carbohydrates include of one or two sugars (monosaccharides or disaccharides) combined win a simple molecule such as fructose, lactose, maltose, sucrose, glucose, galactose and ribose. Complex carbohydrates are made up of sugar molecules that are strung together in long and complex chain such as glycogen, starch, and cellulose, which are multiple glucose molecules [20]. Some carbohydrates have an effect on diabetes as enzyme inhibitors [12]. The types of carbohydrates and compounds that could act as hypoglycemic agents are shown in Fig. 3.

Polysaccharides, the primary constituents of carbohydrates in nature, are monosaccharides and can be derived from plants, algae, animals, fungi, and microorganisms [20–22]. Among complex carbohydrates, polysaccharides are particularly valued for their significant health benefits. Specific polysaccharides possess unique biological activities; for instance, heparin in the human body exhibits anticoagulant properties [23]. Polysaccharides are extensively utilized across various fields, including the food industry, pharmaceuticals, drug delivery systems, and medicine, where they serve as immunoregulatory, anti-tumor, antiviral, anti-inflammatory, antioxidant, and hypoglycemic agents [24]. Research has suggested that polysaccharides can mitigate diabetes by enhancing gastrointestinal viscosity, promoting satiety, facilitating colon fermentation, and reducing gastrointestinal inflammation [25]. Furthermore, the anti-diabetic properties of specific polysaccharides have been attributed to their enzyme inhibition mechanisms [26].

**Fig. 4:**
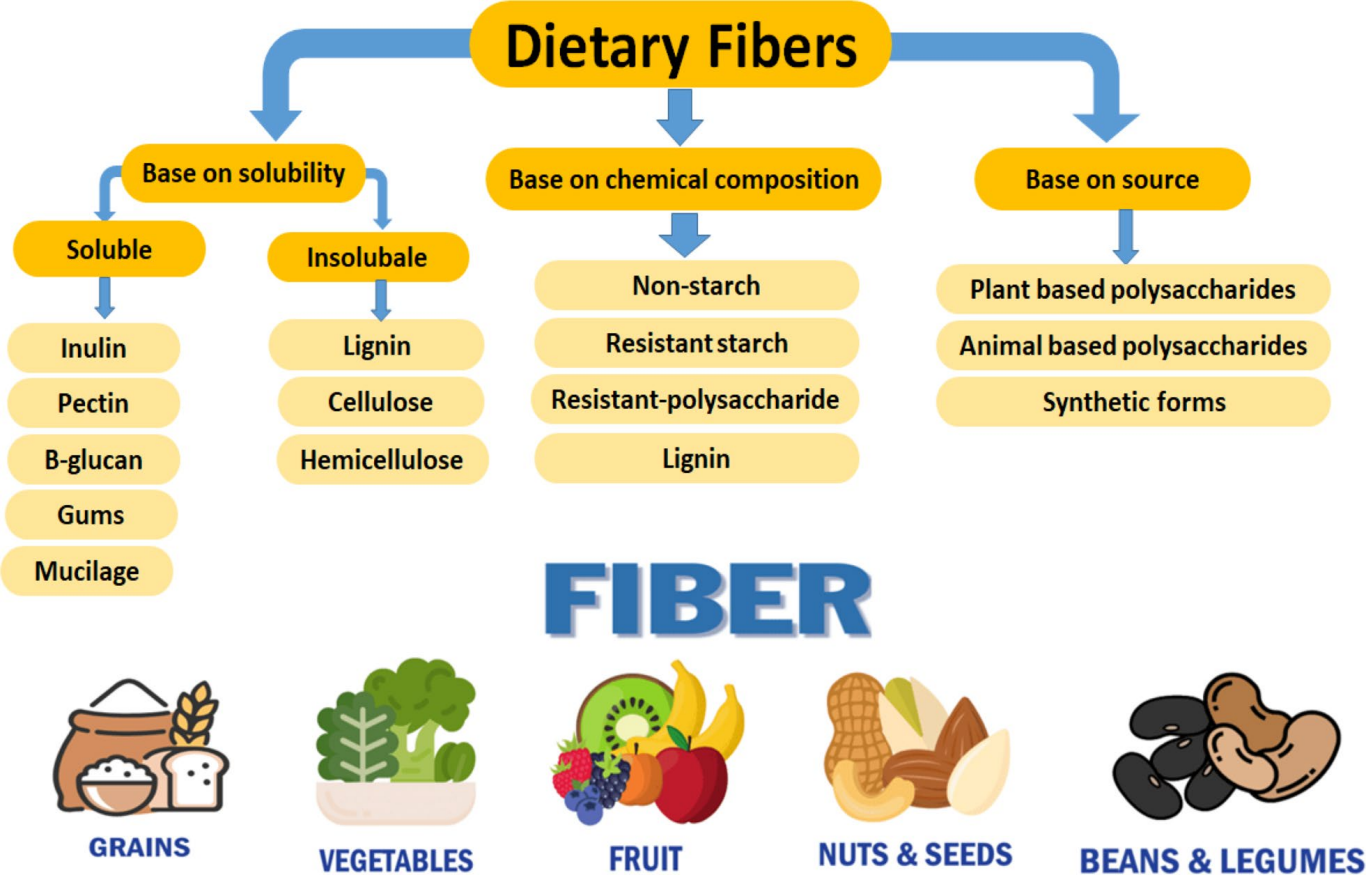
Classification of dietary fibers based on solubility, chemical composition and source

Dietary fibers (DFs), a specific type of polysaccharide, hold considerable value for their physiological benefits, including lowering the risk of diseases such as cancer, obesity, asthma, diabetes, and cardiovascular conditions. Recognized as the seventh essential nutrient for humans, alongside protein, fat, carbohydrates, vitamins, water, and minerals, DF exerts its effects by modulating various physiological processes [27]. Classification of DFs based on solubility, chemical composition and their source are shown in Fig. 4.

Based on chemical composition, DFs are classified as non-starch polysaccharides, resistant oligosaccharides, resistant starch, and lignin based on their chemical properties [20]. Non-starch polysaccharides (NSPs), a group of plant-derived polysaccharides, are typically resistant to digestion in the small intestine due to the absence of endogenous enzymes. Experimental and biological research have demonstrated that fibers like agar gum, beta-glucan, and arabinoxylan enhance viscosity and reduce macronutrient hydrolysis by preventing enzyme–substrate interactions [28]. It is well-established that polysaccharides can inhibit α-amylase and α-glucosidase enzymes. Table 1 showed inhibitory activity of polysaccharide by α-amylase and α-glucosidase enzymes.

The results which were presented in Table 1 show that polysaccharides can significantly inhibit α-amylase and α-glucosidase enzymes. Until now, limited research has been conducted on the effect of the polysaccharides in inhibiting the DPP-IV enzyme [49]. Therefore, evaluating the inhibitory effects of polysaccharides on this enzyme is very important and worthwhile. In addition, the evaluation of polysaccharides showed that Fucoidans are sulfated polysaccharides derived from brown algae or some marine invertebrates. They have good anti-diabetic potential. Fucoidan consists of L-fucose, sulfate ester groups, other monosaccharides, uronic acids, and acetyl [26]. Some research showed that the inhibitory mechanism of Fucoidan can be attributed to the electrostatic attraction between anionic sulfate groups in the polysaccharide chain and the enzyme [50]. In addition, Table 1 shows the inhibitory potential of dietary fibers such as Pectin, Beta-glucan, Arabinoxylan, etc. Research on the role of dietary fiber in diabetes management has indicated that it may delay α-amylase activity and inhibit enzymes by encapsulating starch and enzymes. For example, fibers such as guar gum and pectin, which significantly increase viscosity in the small intestine, have reduced postprandial glycemic responses [51]. Although the enzyme inhibitory ability of dietary fibers is considered one of their anti-diabetic mechanisms, other factors affecting the inhibition rate, such as the structural characteristics of polysaccharides, monosaccharide composition, molecular size, degree of esterification, degree of purity, and the test method should also be taken into account [44, 51–53]. Even, it has been reported that the sulfation of the barley polysaccharide significantly raises the enzyme inhibitory activity with an increase in dose and the degree of substitution of the sulfate group [29].

In addition, Table 1 illustrates the significant potential of polysaccharides in inhibiting α-amylase and α-glucosidase enzymes. However, various factors influence their activity against α-amylase and α-glucosidase. Specifically, the presence of functional groups or compounds such as free carboxyl (–COOH) groups, tannins, phytic acid (C₆H₁₈O₂₄P₆), and uronic acid in polysaccharides suppresses these enzymes, directly reducing their activity. Polysaccharides also adsorb onto starch, interfering with hydrolysis by α-amylase and α-glucosidase. Therefore, when assessing the inhibitory effects of polysaccharides on these enzymes, these factors must be considered, necessitating further research in this area. Additionally, multiple parameters, including molecular weight, glycosidic linkages, extraction methods, monosaccharide composition, viscosity, hydrophilicity, and gel-forming properties, modulate the inhibitory activity of polysaccharides [54]. In a review article, the relationships of structure-hypoglycemic effect of some polysaccharides are also discussed [55]. However, the structure-hypoglycemic activity relationship of polysaccharides, especially monosaccharide composition, molecular weight, higher structure, and type and location of glycosidic bond, is still unclear, which needs continuous attention to further reveal the deeper relationship between them. Additionally, the results showed that complex carbohydrates positively affect the inhibition of α-amylase and α-glucosidase enzymes. Therefore, it is possible to hope for the production of anti-diabetic food products containing polysaccharides. However, research on the specific effects of polysaccharides on the inhibitory activity of these enzymes remains limited and requires further exploration. Also, it should be noted that polysaccharides extracted from other food sources should be further evaluated in terms of clinical, animal, safety, and toxicity research.

### Liposoluble components

**Fig. 5:**
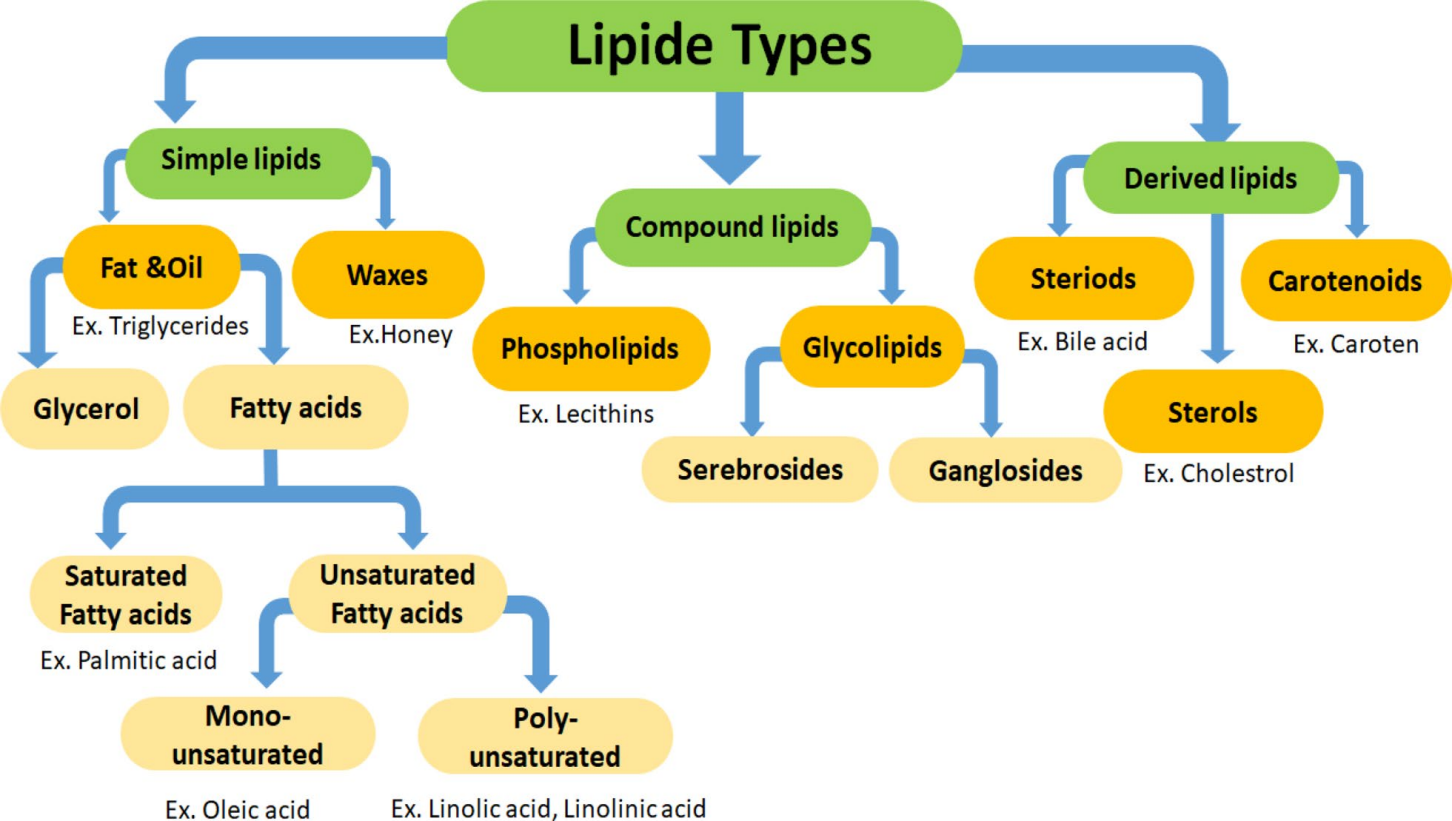
Classification of lipids and liposoluble components that could act as hypoglycemic agents

Lipids are organic compounds essential for the structure and function of living cells. They are critical in forming biological membranes, providing energy, and supporting cell growth. Lipids are categorized into simple and compound lipids, depending on the presence or absence of fatty acids in their structure. Oils and liposoluble compounds also strongly hypoglycemic activity [13]. The classifications of lipids and liposoluble components that could act as hypoglycemic agents are illustrated in Fig. 5.

Liposoluble components are a vital source with significant potential to inhibit α-amylase and α-glucosidase enzymes, making them promising candidates for therapeutic applications and functional food development [56].

**Fig. 6:**
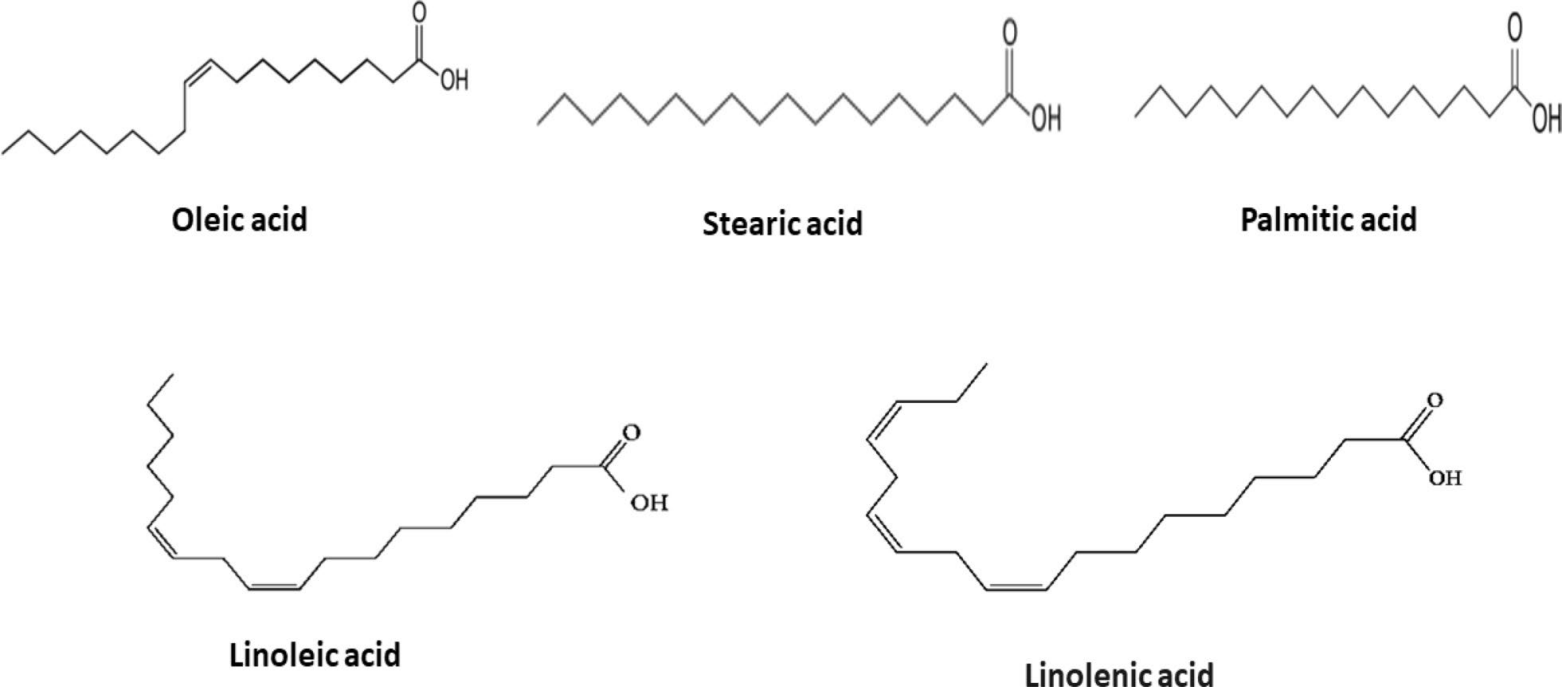
Chemical structure of common FFAs constituents extracted from seed oils hypoglycemic agents

Fatty acids, the simplest lipids, are long hydrocarbon chains with a single carboxyl group. The primary constituents of seed oils include free fatty acids (FFAs), categorized as saturated fatty acids (SFAs), monounsaturated fatty acids (MUFAs), and polyunsaturated fatty acids (PUFAs) [13]. Plants and vegetables are excellent sources of fatty acids. FFAs and their derivatives have garnered attention for their broad spectrum of carbohydrate digestive enzyme inhibition and relatively low toxicity. In Fig. 6, chemical structure of common FFAs constituents extracted from seed oils as natural inhibitors are presented.

Specific research has highlighted unsaturated fatty acids' (UFAs) inhibitory potential. For instance, 7(Z)-octadecenoic acid and 7(Z),10(Z)-octadecadienoic acid, isolated from the body wall of *Stichopus japonicus* (sea cucumber), exhibited α-glucosidase inhibitory activity [57]. Similarly, palmitic acid extracted from the crude extract of *B. pyramidatum* demonstrated α-glucosidase inhibition with an (IC_50_ = 0.237 mM) [58]. This research suggests that the inhibitory efficiency of UFAs increases with the number of double bonds, indicating that the presence of double bonds plays a critical role in α-glucosidase inhibition [13]. Additionally, structure–activity relationship research reveals that UFAs and phosphate groups in glycerides are essential structural elements for inhibitory activity [59].

**Fig. 7:**
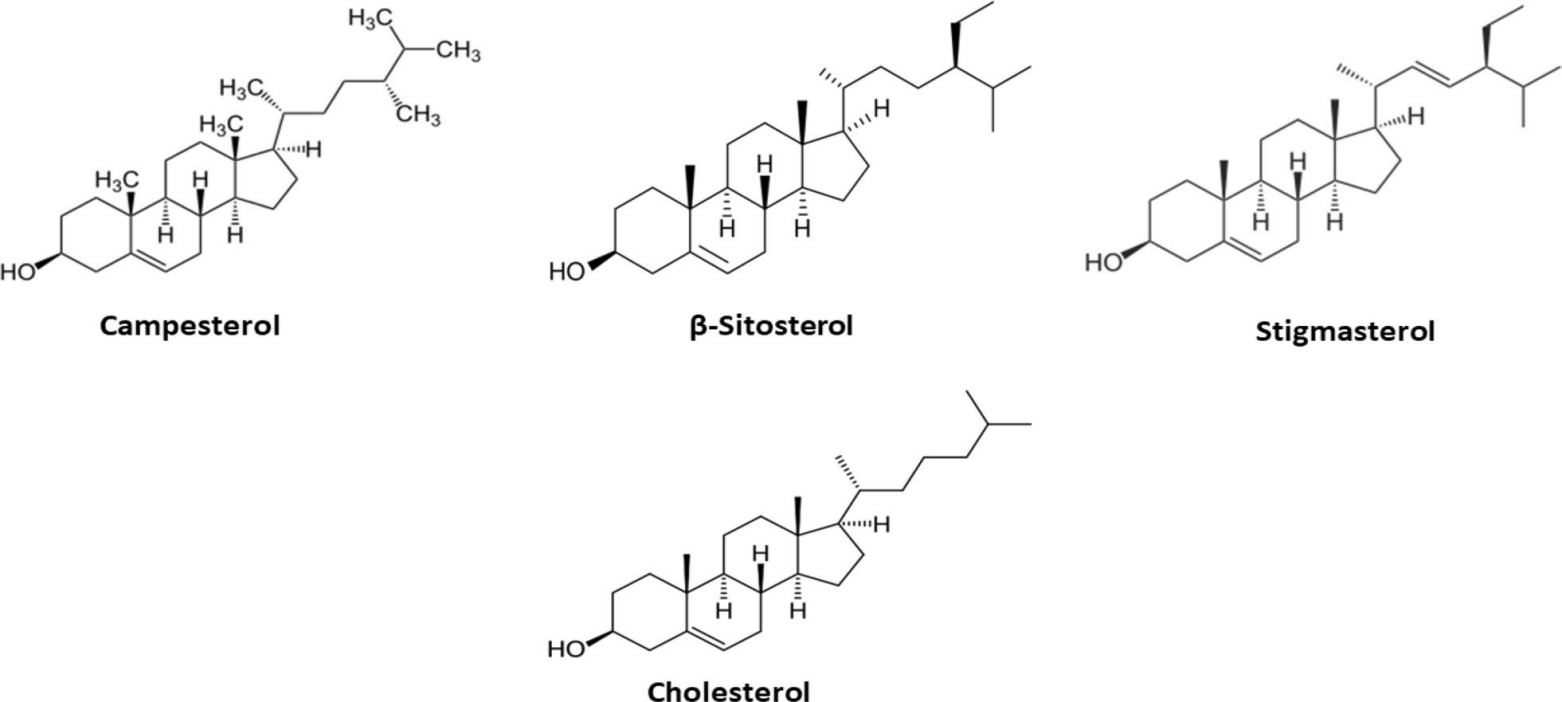
Chemical structure of common phytosterols and cholesterol

Phytosterols, another group of plant-derived liposoluble compounds, structurally resemble cholesterol and steroids and are abundant in flax seeds, sesame, peanuts, almonds, hazelnuts, and wheat germ. Phytosterols compete with cholesterol for intestinal absorption, thereby lowering blood cholesterol levels. The most common phytosterols include campesterol, beta-sitosterol, and stigmasterol. In Fig. 7, chemical structure of these phytosterols in comparison with cholesterol are presented.

Plant-based diets are particularly rich in phytosterols, with nuts, legumes, and seeds providing the highest concentrations. Although fruits and seeds contain lower levels (1–50 mg/100 g), they are still significant sources [13]. Sitosterol from *Chrozophora plicata* showed strong α-glucosidase inhibitory activity (IC_50_ = 0.290 mM); in contrast, stigmasterol demonstrated anti-diabetic effects by inhibiting α-glucosidase in rats [60]. Ergosterol also exhibited α-glucosidase inhibition with an (IC_50_ = 0.839 mM). Other fat-soluble compounds, such as carotenoids and tocopherols, have also been recognized for their anti-diabetic potential to inhibit α-glucosidase [13].

Lipids derived from cereals have been identified as natural enzyme inhibitors (Table 2). Furthermore, evidence suggests that fatty acids, saponins, and terpenes found in fruits, vegetables, and mushrooms contribute to α-amylase and α-glucosidase inhibitory activities in vitro [61]. Despite these findings, many lipid-soluble inhibitors remain unexplored. Future research should investigate the interaction mechanisms between these liposoluble inhibitors and their target enzymes. Examples of lipid-soluble inhibitors studied thus far are summarized in Table 2.

### Phytochemical compounds

**Fig. 8:**
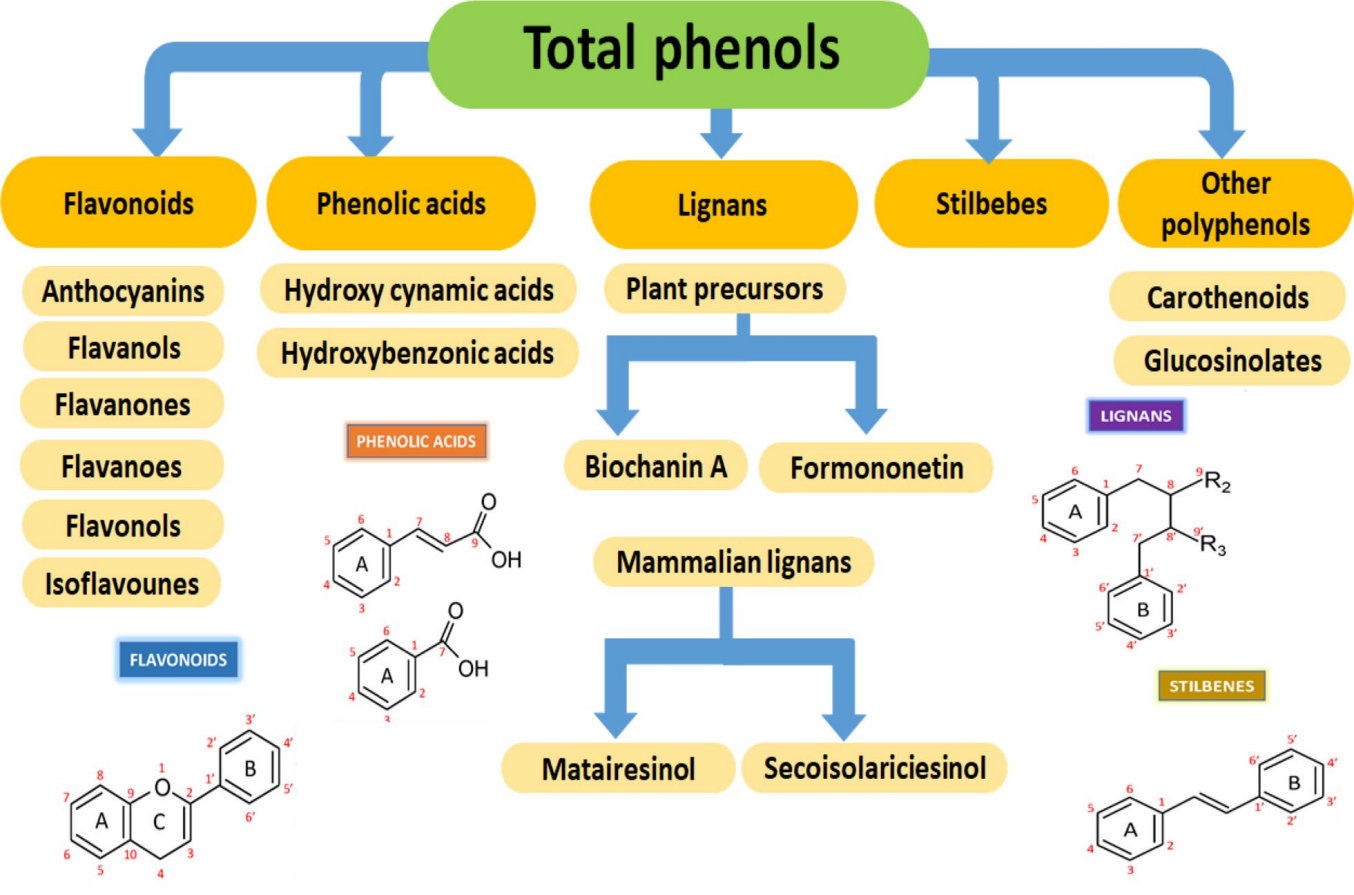
Classification of polyphenols that could act as hypoglycemic agents

Phytochemicals are natural compounds that contribute to plants' color, taste, and aroma and are believed to be the primary agents behind their medicinal properties and health benefits. These compounds are categorized according to their chemical structure [69]. Obviously evaluation of phytochemical compound has proven a very useful way of obtaining antidiabetic therapeutic agents. In this way, the classification of phytochemicals that could act as hypoglycemic agents are presented in Fig. 8.

Phytochemicals include compounds containing phenol (phenolic compounds), especially flavonoids and plant estrogens (phytoestrogens), glucosinolates, and other compounds, including plant-derived sterols (phytosterols) [70]. Various biological phenolic compounds containing one or more aromatic rings are found naturally in natural foods, especially plant compounds; polyphenols are compounds of many phenolic units. Phenols have different sub-categories [71]. Phenolic compounds have exhibited substantial potential in inhibiting α-amylase, α-glucosidase, and DPP-IV enzymes. Secondary plant metabolites, such as polyphenols, are not essential for human or plant growth and development but are often concentrated in plants' leaves and outer parts. Polyphenols possess significant antioxidant and anti-inflammatory properties, which protect against chronic conditions like cancer, cardiovascular diseases, and metabolic disorders, including diabetes, hypertension, and obesity [13]. Table 3 summarises the effects of phytochemical compounds in inhibiting α-amylase, α-glucosidase, and DPP-IV enzymes across various groups of plants, fruits, and vegetables.

According to Table 3, phenolic compounds influence enzyme activity by altering enzyme conformation or stability [96]. Previous studies also showed that phenolic compounds regulate T2D by inhibiting carbohydrate-digesting enzymes [97]. The specific effects of phenol–enzyme interactions depend on factors such as the phenolic compound's structure, concentration, and the enzyme's biochemical properties. These compounds can bind to the active or allosteric sites of the enzyme and affect substrate binding or catalysis. The inhibition may be reversible or irreversible based on the strength and nature of the interaction between the phenolic compound and the enzyme [96]. Furthermore, similar bioactive phenolic compound profiles can help prevent cellular oxidative damage linked to severe diabetic complications. This highlights the importance of focusing on natural compounds and phytochemicals in diabetes control strategies. Numerous investigations have emphasized the pivotal role of phytochemicals in enzyme inhibition [98].

**Fig. 9:**
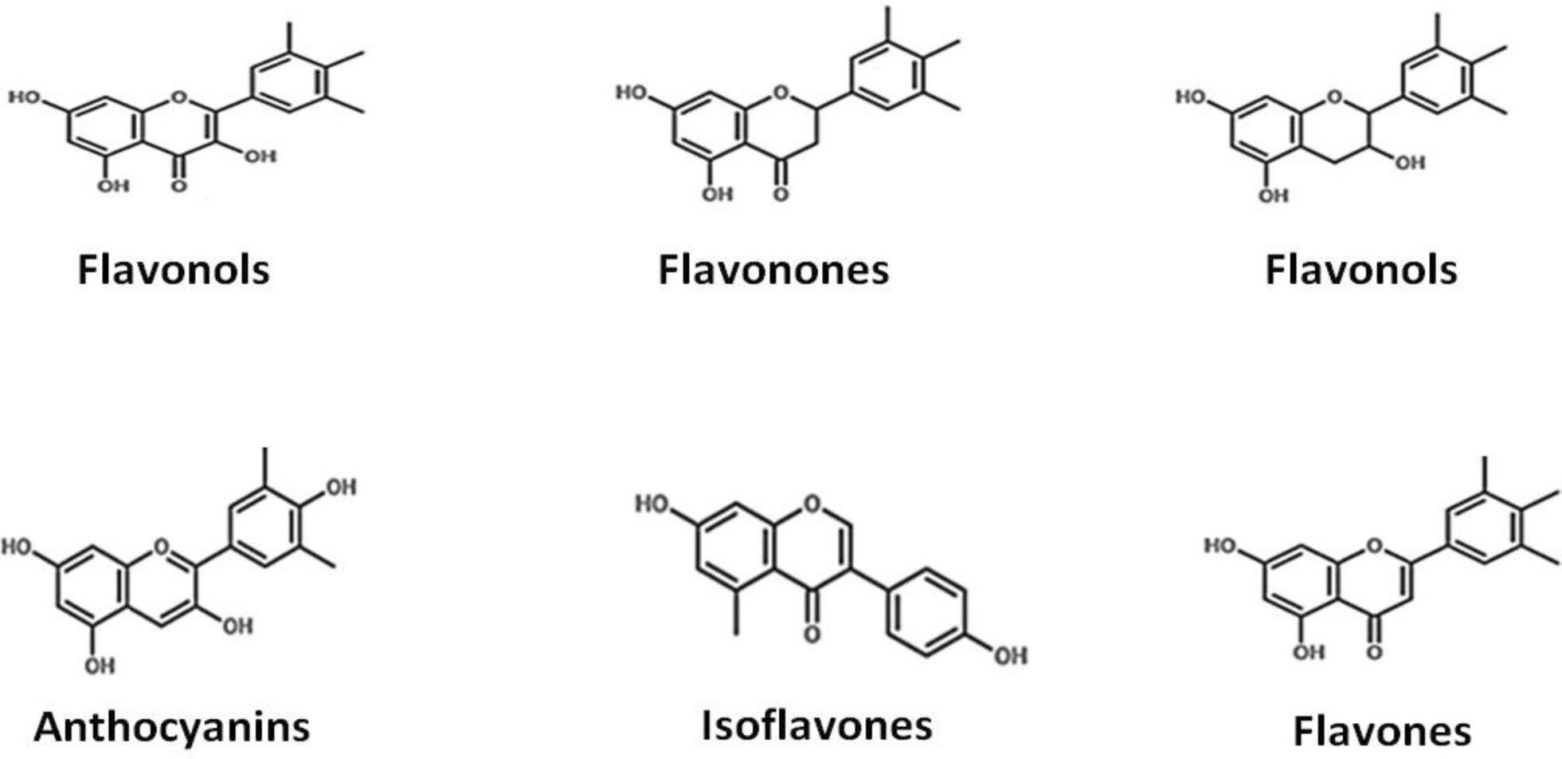
The chemical structure of the more common flavonoids

Flavonoids are abundantly found in plants, vegetables and fruits. Humans usually consume the largest amount of flavonoids through tea, onions and apples.Flavonoids are classified as flavonols, flavanones, flavan-3-ols, anthocyanins, flavones, and isoflavones [20]. The molecular structure of more common and important flavonoids is presented in Fig. 9.

Several studies have established a strong correlation between the anti-diabetic effects of flavonoids and their ability to inhibit α-amylase and α-glucosidase enzymes. Molecular docking analyses further indicate that the inhibitory activity of flavonoids against these enzymes is mediated by hydrogen bonds formed between the –OH groups in the B ring of flavonoids and the catalytic units of the enzymes. Anthocyanins also show considerable potential in enzyme inhibition. Some researchers have reported that these compounds inhibit α-glucosidase more effectively than α-amylase, suggesting a stronger suppressive effect of anthocyanins on starch hydrolysis [54]. The inhibitory effect of anthocyanins is attributed to non-covalent interactions between their hydroxyl groups and other compounds, with evidence suggesting a direct relationship between their hydroxyl content and inhibitory activity [98].

**Fig. 10:**
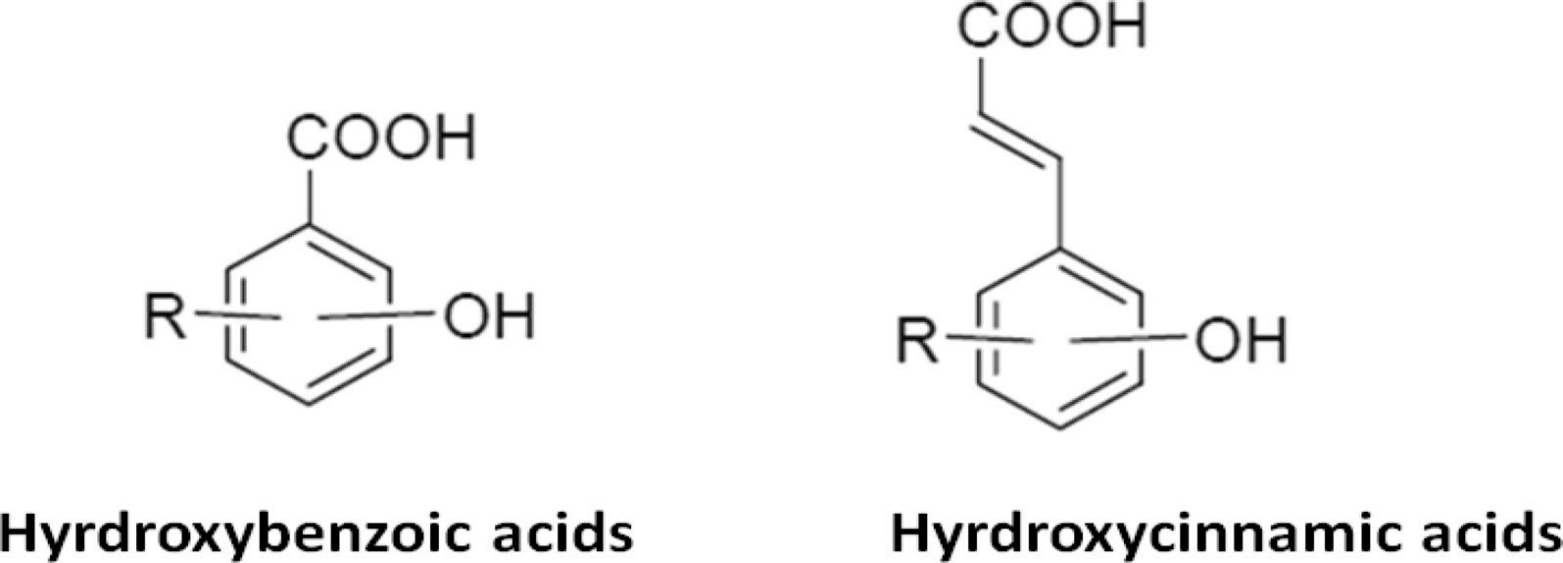
Molecular structure of the most important phenolic acids

Phenolic acids are plant secondary metabolites with carboxylic acid functional group. They are widely distributed in plants. Natural plant phenolic acids mainly include two molecular groups of hydroxycinnamic and hydroxybenzoic acids (Fig. 10).

In some studies, it has been reported that hydroxybenzoic acids, such as vanillic acid and salicylic acid, hardly show inhibitory activity against α-amylase. While hydroxycinnamic acids show good inhibition ability. For hydroxycinnamic acids, the C = C double bonds in the molecular structure with the conjugated carbonyl group are responsible for electron transfer between the benzene and acrylic acid ring segments. As a result, hydroxycinnamic acids can form a highly conjugated system that stabilizes the compounds when they bind to the active site of the amylase enzyme [99]. However, a previous study showed that chlorogenic and other phenolic acids are weak inhibitors of human salivary alpha-amylase and intestinal maltase enzymes [100]. It has also been reported that caffeic acid has a relatively strong inhibitory activity (IC50 = 0.4 mM). However, during investigations, both dehydroxylation and methylation of caffeic acid decreased its inhibitory activity against α-amylase. It was determined for quinic acid (IC50 = 26.5 mM), which was much higher than chlorogenic acids and caffeic acid. Although there are 4 hydroxyl groups in the structure of quinic acid, it does not have a strong conjugated system. Therefore, both conjugated structural features and multiple hydroxyl groups are necessary for hydroxycinnamic acids to exhibit α-amylase inhibition [101]. Some researchers analyzed a series of phenolic acids in different food matrices (for example, coffee, apple, potato, artichoke and plum), as inhibitors of human salivary α-amylase activity and rat intestinal maltase activity and they found that phenolic acids (both as free and linked to quinic acid) were only weak inhibitors of these enzymes [100]. In this regard, some other researchers hypothesized that the inhibition of carbohydrate-digesting enzymes by phenolic acids is not enough to correct postprandial blood sugar and may be the interaction of various factors that determine the food matrix [102]. Therefore, more studies in this field are necessary.

Lignans are polyphenol compounds found in plants. Lignans are mainly found in flax seed. Other sources rich in lignans include fibers such as whole grains, legumes, oilseeds, fruits, and vegetables. It has been reported that the consumption of flax seeds reduces blood glucose and lipid levels, delays glucose absorption after meals, and reduces inflammation and oxidative stress in patients with prediabetes. Lignans can show their effects by different mechanisms, including stimulating insulin secretion, inhibiting pancreatic α-amylase enzyme, increasing insulin sensitivity and improving antioxidant capacity [103].

Stilbenes are other phenolic compounds that not found in abundance in plants, so they are less common in the diet. Stilbenes are mainly found in grapes, berries, peanuts and red wine [104]. Stilbenes have perhaps shown the most scientific and public interest among natural products for controlling obesity and cardiovascular diseases. The Researches in this field over the past two decades have shown that resveratrol has cardioprotective, anti-inflammatory, antithrombotic, anticoagulant, glucose lowering, and neuroprotective effects, but successful clinical trials in humans are few and far between [105]. In a recent study it was shown that the stilbene extracted from seedy bananas imparts inhibit effect on α-glucosidase enzyme [106]. However, more studies in this field are needed.

Carotenoids are important class of natural compounds that occur as pigments in fruits, vegetables, and marine sources. Carotenoids can be divided into two groups, including xanthophylls (eg, lutein and zeaxanthin) and carotenes (eg, α-carotene, beta-carotene, and lycopene). A large number of studies have shown that carotenoids reduce the risk of T2D. It has also been demonstrated that their food consumption has an inverse relationship with HbA1c level. In addition, recent findings have confirmed the protective role of carotenoids such as lycopene, lutein and zeaxanthin against diabetic retinopathy however, it should be noted that the role of carotenoids in the pathogenesis of diabetes is still unclear very well [107]. In vitro studies have shown that carotenoids may play an important role in inhibiting α-amylase and α-glucosidase enzymes in fruit extracts (such as citrus fruits and berries) and vegetables (i.e. pepper) [108].

In general, in the investigation of phytochemical effects on diabetes with the enzyme inhibition method, studies showed that the phenolic compounds found in fruits and vegetables have great potential for diabetes management in controlling hyperglycemia. However, the extraction methods may influence the inhibitory potential of certain plant-derived compounds. Techniques such as maceration, decoction, percolation, infusion, and continuous hot extraction, alongside instrument-assisted methods, including ultrasound-assisted extraction (UAE), microwave-assisted extraction (MAE), and supercritical fluid extraction (SFE), have received increasing attention in recent years. Processing methods may also affect phenolic compound content, influencing enzyme inhibition rates. For instance, filtration and evaporation can modify the phenolic composition [54]. Moreover, variations in inhibitory activity exist among different phytochemical components. Some studies suggest that factors such as the molecular size of phenolic compounds, dietary habits, food matrix composition, gender, and other physiological variables may influence the inhibitory potential of phytochemical [109]. Researchers are actively working to harness these properties by incorporating phytochemicals into food products. For example, these bioactive compounds have been integrated into various food items, including bread, beverages, baked goods, and snacks, as part of strategies to mitigate T2D [110]. The anti-diabetic efficacy of these compounds is influenced by both their concentration and the composition of the food matrix. Additionally, research indicates that bioactive compounds and food processing methods impact the digestibility of starch in foods. It has been observed that some phenolic compounds are prone to evaporation or degradation at elevated temperatures during processing [111]. For instance, the polyphenol content in bread diminishes during baking [112]. Research has reported that berries, pure polyphenols, or their extracts effectively inhibit starch digestion, particularly in liquid food matrices, thereby reducing postprandial glucose absorption. However, their efficacy in starch-heavy foods like bread appears more limited. Similarly, commercial fruit juices enriched with phenolic compounds have shown promise in controlling postprandial glucose levels, especially in liquid foods, facilitating faster sugar absorption than solid starch-based products. In addition, grain-based products such as pasta have been enriched with polyphenols to boost their health-promoting properties, including delayed starch digestion. The influence of polyphenols on starch digestibility is partially attributed to their interactions with carbohydrates. These findings present innovative and promising strategies for diabetes management [113]. However, further research is essential to overcome the challenges associated with the impact of cooking processes and food matrix properties on the efficacy of these bioactive compounds.

### Probiotics

**Fig. 11:**
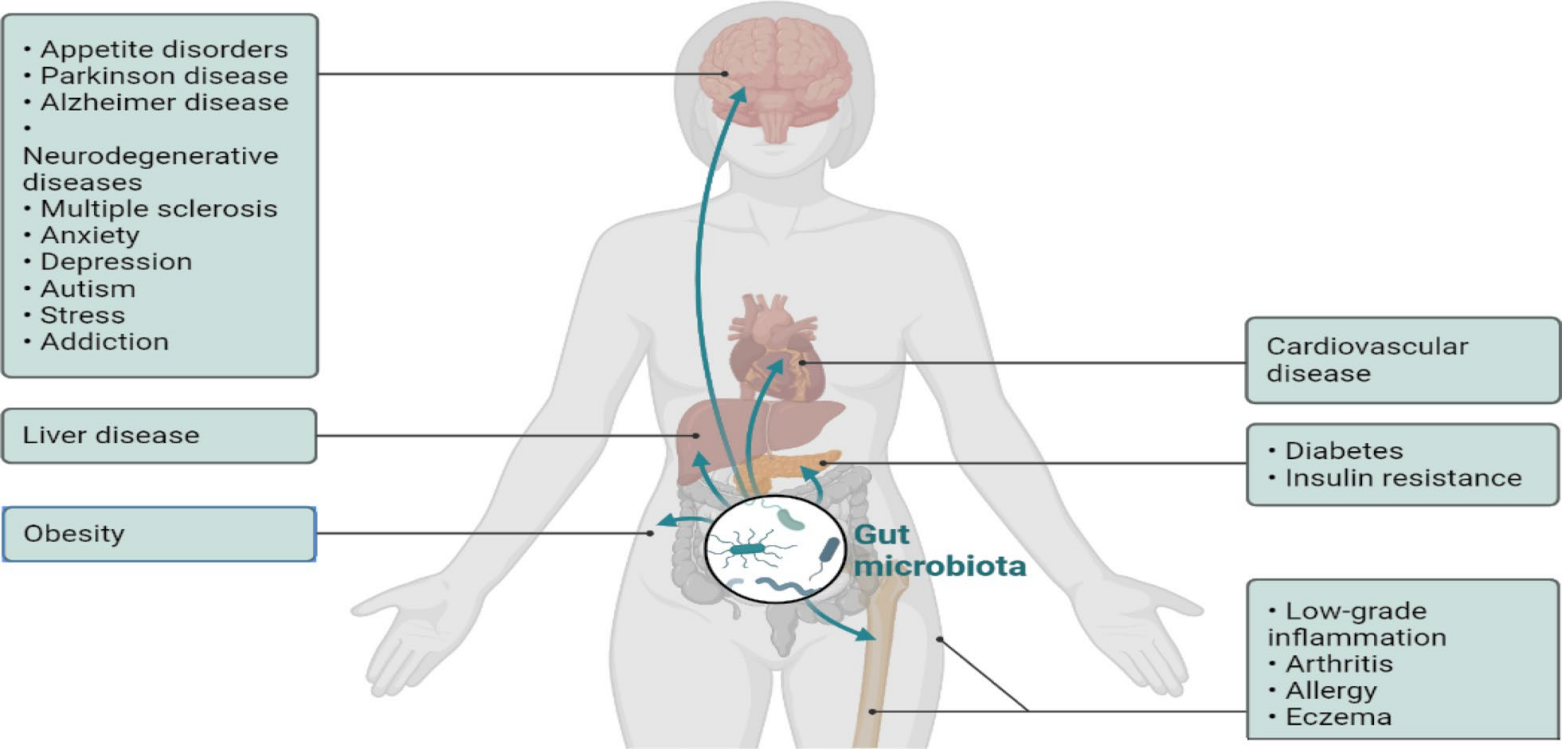
Relationship between gut microbiota and human diseases

In recent years, with the progress of research and investigation on diabetes by researchers, the point that the gut microbiota of each person can play a role in increasing or decreasing diabetes has received more attention because the gut microbiota is entirely different between diabetic and non-diabetic adults. Intestinal microbiota can affect the host's inflammatory pathway and energy metabolism; in other words, changes in intestinal microbiota may affect glucose, fat, and insulin metabolism [114]. Several research studies have revealed a correlation between intestinal microbiota composition and metabolic diseases such as obesity and diabetes [115]. Consequently, one of the most recent and practical approaches to maintaining intestinal microbiological balance involves using probiotics for disease control (Fig. 11).

Probiotics are live microorganisms that confer health benefits to the host when consumed adequately. The primary probiotic strains that positively influence human glucose metabolism predominantly belong to the *Lactobacillus* and *Bifidobacteria* gener*a* [116]. Since managing and controlling diabetes requires a multi-faceted therapeutic approach, probiotic bacteria is a new approach to controlling the disease of diabetes due to their role in the intestinal microbiota. The beneficial effects of probiotics on diabetes have been widely demonstrated in animal research, including a reduction in the Bacteroidetes/Firmicutes ratio, an increase in bacteria producing short-chain fatty acids, a decrease in inflammatory molecules such as tumor necrosis factor-alpha (TNF-α), and an increase in interleukin-1 (IL-1) and interleukin-6 (IL-6), leading to improvements in insulin resistance. In addition, probiotics reduce inflammatory phenotypes, improve beta cell dysfunction, and can benefit the intestinal wall, reduce intestinal permeability, and prevent the transport of bacterial protein lipopolysaccharides [117]. It has also been shown that probiotics can reduce blood glucose and prevent animal stem cell destruction by reducing inflammation [118]. In addition to regulating blood glucose levels through oxidative stress reduction, improved insulin resistance, and increased free fatty acid availability, probiotics have been reported to enhance adiponectin levels [119]. In recent years, consumers have increasingly favored probiotic preparations and functional foods with antidiabetic activity. Lactic acid bacteria strains (LAB) are one of the most probiotic strains. Regarding the role of LAB strains in controlling diabetes, it has been reported that some strains can prevent diabetes by producing different metabolites and inhibiting enzymes [120]. Some fermented by *lactobacillus* strains with anti-diabetic properties. Some researchers' research on foods fermented by *lactobacillus* strains with anti-diabetic properties are presented in Table 4.

Table 4 highlights the significant anti-diabetic potential of probiotics in fermented products. However, factors such as strain safety, health effects, and bacterial viability must be carefully considered when screening anti-diabetic probiotic [119]**.** Most studies focus on dairy-based probiotic formulations, with fermented milk as a particularly suitable matrix for probiotics in enzyme inhibition. These findings emphasize the critical role of fermentation in enzyme inhibition, as probiotic-fermented products exhibit superior anti-diabetic activity compared to non-fermented counterparts. Consistent with these observations, our study also demonstrated anti-diabetic activity in fermented milk samples [2]. However, certain reports suggest minimal inhibitory effects in specific fermented products, potentially due to variations in strain type, inoculation conditions, enzyme specificity, and other influencing factors. Nevertheless, as shown in Table 4 the majority of studies indicate that fermented and functional products effectively inhibit enzymes associated with diabetes. Fermented foods provide higher levels of probiotics and prebiotics than other dietary supplements [119]. Beyond fermented milk, Table 4 identifies fermented fruit juices as an emerging category of functional foods with promising anti-diabetic potential [121, 134]. Studies suggest combining phytochemicals and probiotic fermentation enhances the hypoglycemic effects of these products [135]. Despite the demonstrated inhibitory capacity of probiotics in fermented products, limited research has explored their impact on DPP-IV inhibition. Future studies should investigate this enzyme’s role and functional mechanisms in fermented food matrices. Additionally, while probiotics show strong potential for enzyme regulation, further research is required to elucidate their precise mechanisms in food systems. Extensive molecular and genetic studies are essential for developing probiotic-based products and clarifying probiotic interactions and inhibitory pathways. The complexity of gut microbiota and the scarcity of clinical data further highlight the need for in-depth investigation. Moreover, individual variability and probiotic strain diversity are critical factors influencing their anti-diabetic effects. Recently, postbiotics and paraprobiotics derived from probiotics have gained increasing attention. Reports suggest that postbiotics enhance insulin sensitivity, leading to reduced blood glucose levels and a shorter diabetes progression period [119]**.** These compounds represent promising novel approaches, warranting further comprehensive research.

### Bioactive peptides

**Fig. 12:**
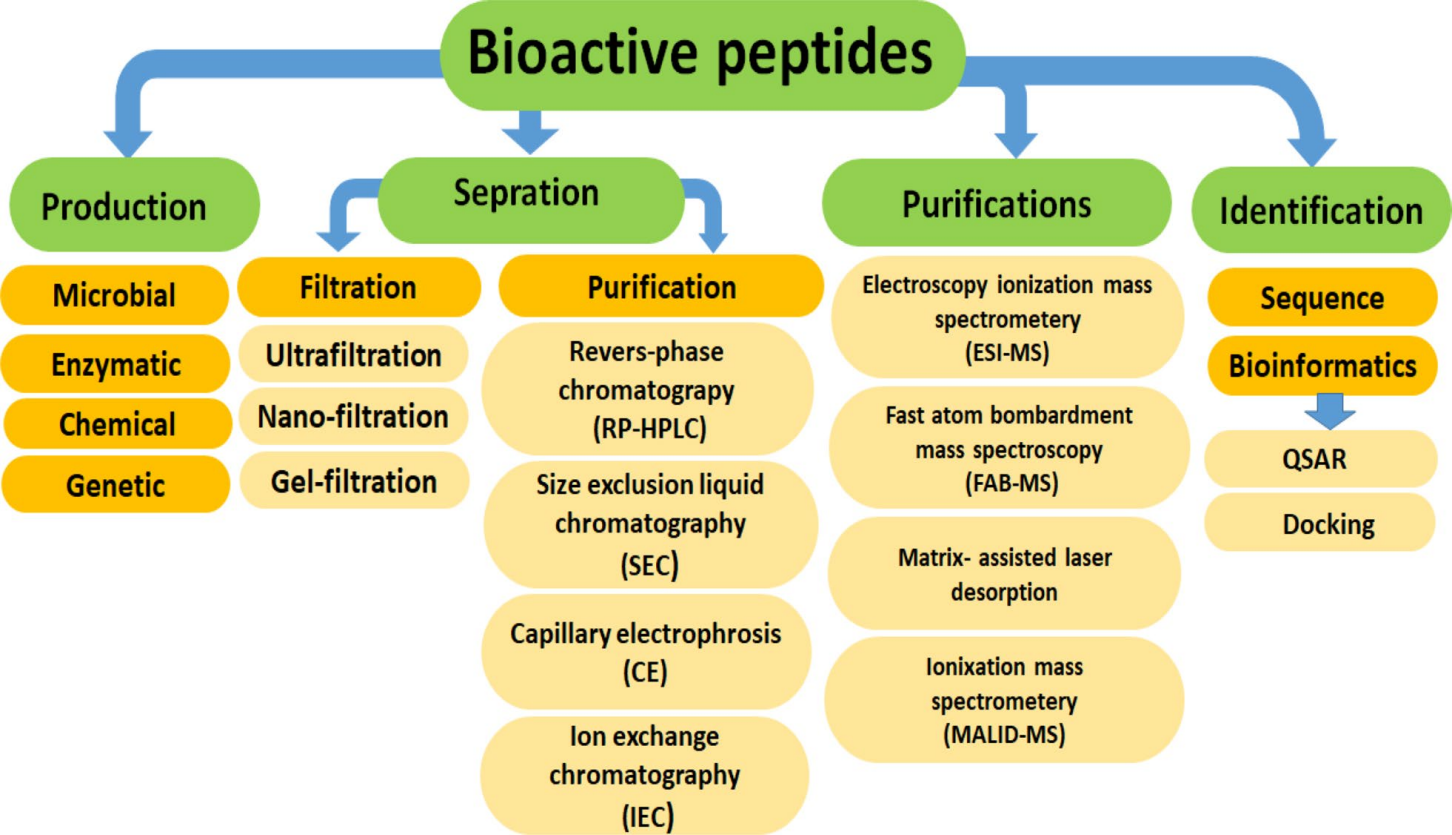
From production procedures up to final identification protocols for bioactive peptides

Bioactive peptides are protein fragments composed of 2 to 20 amino acids, exhibiting diverse biological activities depending on the type and sequence of amino acids [136]. Bioactive peptides and proteins play a vital part in living organisms' metabolic activities and, as a result, in human health [137]. These peptides can be generated through enzymatic hydrolysis using enzymes derived from microorganisms or plants, enzymatic hydrolysis involving digestive enzymes, or fermentation with proteolytic starter cultures [17]. In Fig. 12 an overview of the production process of bioactive peptides from food proteins, including peptide separation, purification techniques, and identification methods is presented.

If the peptide sequence is identified, it can be synthesized through chemical or enzymatic methods or via genetic recombination in bacteria [138]. The production of bioactive peptides through microbial fermentation holds secondary importance but offers distinct advantages. Key benefits of microbial fermentation include selecting specific strains for targeted proteins, optimising bacterial growth temperatures, and precisely controlling fermentation duration [139].

Bioactive peptides produced via microbial fermentation are considered safe, as they are derived from food proteins and involve GRAS (Generally Recognized as Safe) microorganisms [140]. Additionally, this method is more cost-effective than enzymatic hydrolysis, eliminating the need for commercial enzymes and reducing cultivation expenses.

When LAB is utilized, proteases expressed on the cell membrane facilitate simpler purification processes for the bioactive peptides. In addition to bioactive peptides, microbial fermentation generates other biologically active compounds, such as live and dead bacterial cells, exopolysaccharides, and bacteriocins. These additional compounds may contribute to the observed bioactivity, raising uncertainties about whether the effects are exclusively related to peptides or influenced by other components.

Microbial fermentation processes are susceptible to slight variations in parameters such as pH, temperature, and pressure, which can lead to undesirable outcomes. Furthermore, fermentation can introduce unexpected complications and compromise the quality of the final product. Therefore, industrial-scale microbial fermentation requires rigorous monitoring to ensure consistent and standardized product quality [141]. Although producing bioactive peptides using chemical methods is an option, it is often limited by high costs and concerns regarding safety [139].

Recombinant DNA technology enables the production of biologically active peptides, particularly long-chain peptides and proteins. However, this method carries potential risks, as the resulting products may adversely affect the host organism. Moreover, antibacterial peptides exhibit potent antibacterial activity against expression carrier cells and are relatively vulnerable to proteolytic enzymes. To address these challenges, expressing such peptides as chimeric proteins or tandem genes has been proposed to neutralize their inherent toxic properties and enhance their expression levels, thereby mitigating these limitations [138].

In recent years, increasing attention has been directed toward natural alternatives, such as bioactive peptides, for managing T2D. Research has demonstrated the potential to produce bioactive peptides capable of effectively inhibiting enzymes involved in blood sugar regulation [142]. Consequently, developing enzyme-inhibiting peptides has emerged as a promising strategy for managing T2D [143]. Food-derived peptides from food proteins play an important role in regulating glucose homeostasis due to their effects at different levels (for example, regulation of GLP-1 and their capacity to inhibit digestion-related enzymes. In addition, some authors have described peptides as metabolites capable of increasing cholecystokinin (a gut hormone that regulates food intake) [144]. Peptides and amino acids affect body fat reduction, insulin secretion, and glycemic reduction, but more research is needed to explore these mechanisms, including how protein hydrolyzates act as signaling molecules in endocrine cells. Due to the ability of bioactive peptides to regulate intestinal hormones and glucose homeostasis, these compounds are an important research field for controlling diabetes and can positively affect human health and economic levels [144]. Peptides derived from food sources have shown promising evidence to serve as α-amylase, α-glucosidase, and DPP-IV inhibitors, posing in Table 5.

Table 5 highlights that dairy products are significant sources of anti-diabetic bioactive peptides. Research has demonstrated that milk proteins can inhibit enzymes such as α-glucosidase and DPP-IV, which are key contributors to developing T2D. The inhibitory effects of these peptides are mediated through multiple mechanisms, including satiety response, regulation of incretin hormones, modulation of insulin levels, and suppression of carbohydrate-degrading digestive enzymes [17]. Evaluating protein hydrolysates containing bioactive peptides indicates that legumes represent innovative and cost-effective sources of such peptides. Numerous research have confirmed the role of soybean-derived peptides in diabetes management, while various beans have been shown to inhibit digestive enzymes. Quinoa, a pseudo-cereal rich in protein and amino acids, generates anti-diabetic bioactive peptides through enzymatic hydrolysis, primarily by inhibiting digestive enzymes. The anti-diabetic properties of seeds have also been validated by identifying bioactive peptides in nuts. Additionally, flaxseed, rapeseed, sunflower, and sesame proteins offer novel avenues for producing anti-diabetic bioactive peptides. The findings indicate that peptides derived from proteolytic enzyme activity effectively inhibit α-amylase, α-glucosidase, and DPP-IV enzymes and highlight enzymatic hydrolysis as a reliable approach for producing functional peptides with anti-diabetic potential. Some research indicated that the activity of anti-diabetic peptides can be attributed to the proteolytic enzyme used in hydrolysis. Some research reported that the bioactive peptides and fractions (Albumin, Globulin, Prolamin, and Glutelin) obtained from different hydrolysis (Alcalase, Nutrase, Flavourzyme, and Protamax) of rice bran proteins possess different activities in inhibiting α-amylase enzyme [172].

The majority of research conducted thus far has employed proteolytic enzymes such as alcalase, pepsin, trypsin, and chymotrypsin for enzymatic hydrolysis, emphasizing the necessity of exploring other proteases in future investigations. Evidence suggests that peptide molecular weight plays a pivotal role in determining the efficiency of enzyme inhibition. Low molecular weight peptides are particularly effective in suppressing carbohydrate-hydrolyzing enzymes. For example, one investigation demonstrated that the < 3 kDa fraction obtained from pinto bean protein exhibited the highest α-amylase inhibitory activity [173]. Similarly, bioactive peptides with shorter chain lengths have been associated with α-amylase inhibition [131].

The superior efficacy of short-chain peptides may result from their ability to release electrons, thereby enhancing the inhibitory interaction with enzymes [174]. Peptides with low molecular weight can penetrate the enzyme’s active site more readily, disrupting the binding interaction between the protein and the ligand [175]. Furthermore, these peptides may contain more reactive side chains on amino acid residues, which are exposed on their surface, thereby increasing the likelihood of interaction with the enzyme's catalytic region or subsites [175]. Therefore, low molecular weight bioactive peptides could serve as effective agents for diabetes management, provided their absorption, digestion, metabolism, excretion, stability, and potential toxicity are comprehensively evaluated to enhance their bioavailability and stability. The inhibitory mechanism of enzyme activity may be attributed to the differing capacities of various fractions. For instance, specific α-amylase inhibitory peptides have been identified as blockers of the enzyme's catalytic site, explicitly targeting the catalytic Glu and Asp residues. In contrast, others have been shown to inhibit both the catalytic site and the substrate-binding regions [16]. The amino acid composition and peptide sequence are critical factors influencing the inhibitory effects of bioactive peptides. Structural-activity relationship analysis has revealed that the presence and position of specific amino acids significantly impact enzyme inhibition. For instance, DPP-IV inhibitory peptides contain hydrophilic amino acids such as threonine, histidine, glutamine, serine, lysine, and arginine, although the precise role of these hydrophilic residues remains largely unclear. Research has highlighted that a proline residue in the N-terminal region can be an effective precursor for DPP-IV inhibitors. Additionally, amino acids with high molecular weight and aromatic residues, including phenylalanine, tryptophan, tyrosine, and arginine, have been shown to enhance the inhibition of the α-amylase enzyme. Thus, amino-aromatic acids are pivotal in enzyme inhibition due to their interaction with catalytic sites [15]. Furthermore, factors such as the protein source, hydrolysis conditions, protein pretreatment, and the structure and efficiency of protein hydrolysates have been identified as key determinants of bioactive peptide performance [136]. Modifying hydrolysis parameters—including the type of protein and enzyme, the enzyme-to-substrate ratio, and hydrolysis duration—can yield products with distinct functional properties. Therefore, to produce bioactive peptides with the desired functionalities, it is essential to standardize the processes of hydrolysis, separation, and purification [176]. A critical factor influencing the inhibitory properties of peptides is their lower bioavailability and absorption under in vitro conditions compared to physiologically relevant environments. However, to exert anti-diabetic effects within the body, peptides must demonstrate sufficient stability and resistance to endogenous protease enzymes. The discovery of anti-diabetic peptides should extend beyond purification and structural identification to encompass key biochemical properties, including charge, polarity, and solubility, which significantly impact their anti-diabetic activity. In this context, the discovery of anti-diabetic peptides should not only focus on purification and structural identification but also take into account key factors such as charge, polarity, solubility, and other biochemical properties, which play a significant role in evaluating the anti-diabetic activity of peptides [54].

## Limitation and future perspectives

Several limitations emerged during the literature review and assessment of enzyme inhibitory activity, complicating cross-study comparisons.

**Assay methodology:** Substantial variability in assay protocols remains a significant challenge, as methodological differences affect IC₅₀ values and inhibition mechanisms. The lack of a universally accepted reference method undermines meaningful comparisons. But the diversity of the features of different enzymes prevents unification of assay conditions [177]. In addition, sample-specific characteristics—including purity, food matrix composition, essential oils, plant extracts, or isolated compounds—can influence outcomes. Interactions between inhibitor type, compound matrix, and assay methodology further underscore the need for protocol optimization tailored to compound class and matrix complexity.

**Enzyme source:** The enzyme origin significantly impacts assay performance. Most studies assessing α-amylase inhibition use porcine pancreatic enzymes, whereas yeast-derived enzymes dominate α-glucosidase assays. These selections are driven by cost and availability. However, reliance on non-human enzymes may reduce the translational value of findings [178]. Future research should prioritize human-derived enzymes to improve clinical relevance.

**Substrate type:** Substrate selection also introduces variability. For example, turbidity and preparation method differences affect activity measurements [179]. Standardizing substrate type and preparation will be crucial for improving reproducibility and accuracy.

**Experimental conditions**: Physical, chemical, and microbiological assay conditions strongly influence outcomes, including pH, solubility, molecular weight, and incubation time [177]. Rigorous control and reporting of these parameters are required for valid comparisons and meta-analyses.

**Database accessibility:** Limited accessibility to non-English or region-specific publications hampers comprehensive data extraction. As highlighted in Study Lam et al. [178], this restricts the synthesis of global evidence and emphasizes the need for multilingual inclusion in scientific databases.

**Bioavailability**: Their chemical structure and matrix interactions govern the bioavailability of bioactive compounds. Processing methods such as enzymatic or thermal treatment can modify structural integrity and activity. Greater insight into compound-specific moieties, molecular targets, and metabolic transformations will enhance the prediction of therapeutic efficacy in diabetes management [180]. Therefore, proper identification of active components with their molecular interaction is highly necessary [181].

**Lack of research resources**: A shortage of mechanistic studies limits the understanding molecular interactions underlying enzyme inhibition. Due to insufficient interdisciplinary integration, many in vitro studies lack structural or pathway-level analysis. Addressing this gap requires cross-domain collaboration and expanded use of in silico modeling, molecular docking, and cell-based screening platforms. While numerous compounds exhibit promising antidiabetic potential, safety data for novel agents such as emerging polysaccharides remain limited. Preclinical safety evaluations are critical prior to clinical translation [180].

## Conclusion

This study investigated the effects of natural inhibitors on α-amylases, a-glucosidase, and DPP-IV enzymes in vitro for T2D control. The results showed that some polysaccharides, liposoluble and phytochemical compounds, and bioactive peptides have excellent inhibitory effects. The review of research indicated that fermented foods produced by probiotics can strongly affect enzyme inhibition. These results may become promising for T2D treatment. In this regard, the findings of this research underscore the importance of functional compounds and the development of functional foods in managing and controlling T2D, contributing positively to human health. However, many natural inhibitors are still not known. Future studies require new natural inhibitors and their probable toxicity.

## Data Availability

Not applicable.

## Abbreviations

DPP-IV: Dipeptidyl peptidase-IV
FFAs: Free fatty acids
GIP: Glucose-dependent insulinotropic peptide
GLP-1: Glucagon-like peptide-1
GRAS: Generally recognized as safe
IC_50_: Half-maximal inhibitory concentration
IL1: Interleukin-1
IL6: Interleukin-6
LAB: Lactic acid bacteria strains
MAE: Microwave-assisted extraction
MUFAs: Monounsaturated fatty acids
PUFAs: Polyunsaturated fatty acids
SFAs: Saturated fatty acids
SFE: Supercritical fluid extraction
TNF-α: Tumor necrosis factor-alpha
T2D: Type II diabetes
UAE: Ultrasound-assisted extraction
UFAs: Unsaturated fatty acids' inhibitory potential
